# CD40 Activity on Mesenchymal Cells Negatively Regulates OX40L to Maintain Bone Marrow Immune Homeostasis Under Stress Conditions

**DOI:** 10.3389/fimmu.2021.662048

**Published:** 2021-05-18

**Authors:** Barbara Bassani, Claudio Tripodo, Paola Portararo, Alessandro Gulino, Laura Botti, Claudia Chiodoni, Elena Jachetti, Niccolò Bolli, Marilena Ciciarello, Korinna Joehrens, Ioannis Anagnostopoulos, Il-Kang Na, Antonio Curti, Mario P. Colombo, Sabina Sangaletti

**Affiliations:** ^1^ Department of Research, Fondazione IRCCS Istituto Nazionale Tumori, Milan, Italy; ^2^ University of Palermo School of Medicine, Palermo, Italy; ^3^ Department of Oncology and Hemato-Oncology, University of Milan, Milan, Italy; ^4^ Hematology, Fondazione Cà Granda IRCCS Policlinico, Milan, Italy; ^5^ Department of Experimental, Diagnostic and Specialty Medicine—DIMES, Institute of Hematology “Seràgnoli”, Bologna, Italy; ^6^ Charité-Universitätsmedizin Berlin, Institute of Pathology, Berlin, Germany; ^7^ Institute of Pathology, University of Wuerzburg, Wuerzburg, Germany; ^8^ Department of Hematology, Oncology and Tumor Immunology, Charité-Universitätsmedizin, Berlin, Germany; ^9^ Berlin Institute of Health (BIH), Berlin, Germany; ^10^ Experimental and Clinical Research Center (ECRC), Berlin, Germany

**Keywords:** B-cell development, CD40, OX40L, mesenchymal cell, bone marrow transplantation

## Abstract

**Background:**

Within the bone marrow (BM), mature T cells are maintained under homeostatic conditions to facilitate proper hematopoietic development. This homeostasis depends upon a peculiar elevated frequency of regulatory T cells (Tregs) and immune regulatory activities from BM-mesenchymal stem cells (BM-MSCs). In response to BM transplantation (BMT), the conditioning regimen exposes the BM to a dramatic induction of inflammatory cytokines and causes an unbalanced T-effector (Teff) and Treg ratio. This imbalance negatively impacts hematopoiesis, particularly in regard to B-cell lymphopoiesis that requires an intact cross-talk between BM-MSCs and Tregs. The mechanisms underlying the ability of BM-MSCs to restore Treg homeostasis and proper B-cell development are currently unknown.

**Methods:**

We studied the role of host radio-resistant cell-derived CD40 in restoring Teff/Treg homeostasis and proper B-cell development in a murine model of BMT. We characterized the host cellular source of CD40 and performed radiation chimera analyses by transplanting WT or *Cd40-KO* with WT BM in the presence of T-reg and co-infusing WT or - *Cd40-KO* BM-MSCs. Residual host and donor T cell expansion and activation (cytokine production) and also the expression of Treg fitness markers and conversion to Th17 were analyzed. The presence of *Cd40+* BM-MSCs was analyzed in a human setting in correlation with the frequency of B-cell precursors in patients who underwent HSCT and variably developed acute graft-versus-host (aGVDH) disease.

**Results:**

CD40 expression is nearly undetectable in the BM, yet a *Cd40-KO* recipient of WT donor chimera exhibited impaired B-cell lymphopoiesis and Treg development. Lethal irradiation promotes CD40 and OX40L expression in radio-resistant BM-MSCs through the induction of pro-inflammatory cytokines. OX40L favors Teff expansion and activation at the expense of Tregs; however, the expression of CD40 dampens OX40L expression and restores Treg homeostasis, thus facilitating proper B-cell development. Indeed, in contrast to dendritic cells in secondary lymphoid organs that require CD40 triggers to express OX40L, BM-MSCs require CD40 to inhibit OX40L expression.

**Conclusions:**

CD40+ BM-MSCs are immune regulatory elements within BM. Loss of CD40 results in uncontrolled T cell activation due to a reduced number of Tregs, and B-cell development is consequently impaired. GVHD provides an example of how a loss of CD40+ BM-MSCs and a reduction in B-cell precursors may occur in a human setting.

## Introduction

Bone marrow (BM) is the primary lymphoid organ and is dependent upon hematopoietic development; however, it is also capable of activating secondary lymphoid organs. The BM is the site where lymphoid precursors are generated, and it is also a site where mature T cells recirculate and home. Within the BM, T lymphocytes display a peculiar CD8-to-CD4 ratio (in favor of the former) and are dispersed throughout the BM stroma and parenchyma or condensed in lymphoid tissues surrounding blood vessels. Such lymphoid tissues can expand during infection and inflammation, thus suggesting the establishment of an effective immune response in this organ (1). Differentiated dendritic cells that are present within these pseudo-follicles can potentially process and cross-present antigens to prime T cells and generate effector T cells capable of destroying antigen-expressing cells. This renders BM a potential source of effector T cells. Activated T-cells produce a variety of inflammatory mediators (i.e., IFN -, TNF-, and IL-1) that can alter hematopoiesis in a manner that favors myeloid cells at the expense of lymphoid cells (2). Based on this, it is important to promptly restore immune homeostatic conditions. The peculiar enrichment in Foxp3+ regulatory T-cells (Tregs) that represent up to 30%–40% of total CD4 T cells to levels exceeding 3- to 4-fold the fraction of Tregs in other lymphoid organs is in agreement with the re-establishment of immune homeostasis and proper hematopoietic development. A tight link between Tregs and lymphoid cell development also exists for the B-cell lineage. Indeed, B-cell lymphopoiesis is impaired in Treg-depleted mice, as Tregs control the physiological production of IL-7 that is required for normal B-cell lymphopoiesis (3). The negative effect of IFNα on B-cell lymphopoiesis (4, 5) suggests that the inhibition of effector T-cells by Tregs could represent another mechanism that is involved in restoring B-cell lymphopoiesis by Tregs. Therefore, regulation of Treg development and activity within the BM is an important aspect of the physiology of this organ.

In the periphery, Treg development depends upon TGF-β and IL-2, while in the thymus, IL-2 is dispensable due to coexisting cytokines (IL-2 and IL15) acting on IL-2Rβ (6). Additionally, the costimulatory CD40/CD40L axis and the OX40/OX40L axis contribute to peripheral Treg (7) homeostasis through divergent functions. CD40 is required to maintain the proper Treg number in peripheral blood and secondary lymphoid organs (SLO) *via* IL-2 (8), while the engagement of OX4O blocks the generation of new Tregs and the suppressive function of Tregs (9, 10) through a reduction in the secretion of the cytokine IL10 (11).

In contrast to SLO, the regulation of Treg homeostasis within the BM remains largely uncharacterized. Bone marrow mesenchymal stromal cells (MSCs) nurse hematopoietic stem cells and progenitor cells through the organization of specialized niches sustaining quiescence/self-renewal and also promote cell differentiation toward myeloid and lymphoid lineages (12). In addition to supporting HSC function, BM-MSCs exert immune-regulatory functions, where they can inhibit the proliferation of T and B lymphocytes and natural killer (NK) cells, impair dendritic cell (DC) maturation (13), and promote Treg differentiation from naïve T cells *in vitro via* ICOSL expression (14). Such regulatory activity of BM-MSCs toward Tregs is particularly relevant within the BM, where these two cell types co-exist within the same niches to facilitate proper hematopoietic development through their cross-regulation.

In addition to soluble factors such as IL-7, other structural ECM-related glycoproteins can participate in these regulatory activities. We have previously demonstrated that the removal or down-modulation of the matricellular protein SPARC in BM-MSCs in BMT settings negatively affected both B cell development (15) and Treg composition (in favor of Teff) (16), thus highlighting the close physical and functional link between BM-MSCs and Tregs toward the proper B-cell lymphopoietic BM function. Although Tregs control IL-7-mediated BM-MSC support to B-lymphopoiesis (3), the molecules expressed by BM-MSCs that are responsible for BM Treg regulation remain undefined. In this study, we reveal the necessary role of stress/radiation-induced CD40 in radio-resistant BM-MSCs to restore B-cell lymphopoiesis and T-cell homeostasis in the BM niche in the context of BM transplantation.

## Materials and Methods

### Patients

This study included bone marrow biopsies from 12 adult patients with acute leukemia who underwent allo-HSCT at the Charité University Hospital.

The patient clinical characteristics and GVHD prophylaxis are summarized in Additional File 1: **Supplementary Table 1**.

The study was approved by the Charité-Berlin local ethics committee. Informed consent was obtained from all patients. This study was conducted in accordance with the Declaration of Helsinki.

### Mice

BALB/cAnNCrl (CD90.1 and CD90.2) and C57BL/6 mice were purchased from Charles River Laboratories (Calco, Italy). *Tnfrsf5* (*Cd40*)-KO mice on a BALB/c (B/c) or C57BL/6 (B6) background were already available in our laboratory or were obtained from Dr. Dellabona Paolo (San Raffaele Hospital, Milan, Italy) and Prof. Bronte Vincenzo (University of Verona, Verona, Italy), respectively. CxB6F1 (*Cd40*)-KO mice were obtained by crossing the *Cd40*-KO mice on a B6 background with *Cd40*-KO on a B/c background. Foxp3-GFP knock-in mice were originally obtained from Adorini L. (intercept Pharmaceuticals). All experiments involving animals described in this study were approved by the Italian Ministry of Health (authorization number n. 601/2019-PR).

### BMT and aGVHD Mouse Experiments

Canonical BMT experiments were performed by transplanting 2 × 10^5^ lin- cells from donors into lethally irradiated WT and *Cd40*-KO mice as previously described (17). MHC-mismatched BMT (GVHD) was performed by infusing lethally irradiated B/c mice with 2 × 10^6^ T-cell-depleted BM cells from C57BL/6 (B6) mice in the presence or absence of Teff cells (2 × 10^5^) from the same donors. Control mice were obtained by infusing B/c recipients with 2 × 10^6^ T-cell-depleted BM cells and 2 × 10^5^ Teff from donor B/ c mice.

T-cell-depleted BM was obtained by flushing the BM cells from donors and incubating them with α-CD5 (Ly-1) microbeads purchased from Miltenyi. Unlabeled cells that passed through the magnetic column were used for the transplantation experiments. Mice were visually inspected daily using an aGVHD scoring system that measures items related to the known clinical signs of GVHD, including posture, activity, fur texture, skin integrity, diarrhea, and weight loss every 3 days.

### Flow Cytometry

The composition of the B cell compartment in the BM and spleens of BM chimeras was analyzed as previously described (15). Notably, B-cell development in the BM was classified into sequential subsets designated as Fractions A, B, C, C’, D, E, and F as originally described by Hardy et al. (18).

Surface staining was performed in phosphate-buffered saline (PBS) supplemented with 2% fetal bovine serum (FBS) for 30 min on ice. Foxp3 intracellular staining was performed according to the manufacturer’s instructions (eBioscience). Prior to IFNγ staining, the cells were stimulated *in vitro* for 4 h at 37°C using Cell Stimulation Cocktail plus protein transport inhibitors (eBioscience).

All antibodies are listed in **Supplementary Table 2**. Flow cytometry data were acquired on an LSRFortessa (Becton Dickinson) and analyzed using FlowJo software (version 8.8.6, Tree Star Inc.).

### Isolation and Culture of Murine BM-MSCs

Murine BM-MSC cultures were obtained from the trabecular fraction of femurs and tibias of WT and *CD40-*KO mice. Briefly, the cellular fraction of the femurs and tibias was washed out, and the compact bone was incubated with collagenase I (1 mg/ml) for 1 h at 37°C. After enzyme digestion, the bone suspension was passed through a 70-mm filter mesh to remove any bone spicules and large tissues. Cells were seeded in complete medium at a density of 25 × × 10^6^ cells/ml. Floating cells were removed every 3-4 days. Adherent cells were phenotypically characterized using CD31, CD45, CD34, Ter119, CD44, Sca, and c-Kit antibodies. *In vitro* and *in vivo* experiments involving murine BM-MSCs were performed using cells that were between the 2nd and 5th passages.

### MSC Stimulation and Treatment With Anti-CD40 Monoclonal Antibodies

To evaluate *cd40* modulation upon cytokine stimulation, MSCs isolated from WT or Cd40-KO mice were treated for 24 h, 4 days, or 7 days with IFN-γ (10 ng/ml), TNF-α (50 ng/ml), IL-1β (10 ng/ml), GM-CSF (40 ng/ml), IL-6 (40 ng/ml), IL-17 (100 ng/ml), and TGF-β (5 ng/ml). To evaluate *Ox40l* expression upon cytokine stimulation, MSCs isolated from WT or *Cd40-KO* mice were treated with IFN-γ (10 ng/ml) and TNF-α (50 ng/ml). For the experiment using agonistic CD40 monoclonal antibody, MSCs or DCs isolated from WT mice were stimulated for 2 h with IFN-γ (10 ng/ml) and TNF-α (50 ng/ml) and then treated for 24 h with anti-mouse CD40 (Clone: FGK45.2) monoclonal antibody or isotype control (5 μg/ml). All cytokines were obtained from Peprotech.

### Cytotoxicity Assay

MSCs (isolated from B/c mice and expanded *in vitro*) *in vitro* were treated with IFNγ for 24 h to promote their expression of CD40, and they were then labeled with CFSE (BD Biosciences, cat no. 565082), washed, and incubated for 4 h with splenocytes that were freshly isolated from aGVHD or control mice. The ratio was 1.25 × 10^6^ splenocytes to 5 × 10^4^ MSCs (50:1). Control conditions were represented by MSCs alone and without splenocytes to evaluate spontaneous cell death or by MSCs incubated with splenocytes from a naïve mouse. After 4 h, the cells were labeled with BD Horizon™ Fixable Viability Stain 780 (cat. no. 565388) according to the manufacturer’s instructions and acquired by BD LSR Fortessa. Specific MSC lysis was determined as follows: % specific lysis = 100 × lrb% of dead target % spontaneous dead target)/(100% spontaneous dead target), as described previously (19).

### Statistical Analysis

All statistical analyses were performed using Prism 8 software (GraphPad Software). The statistics applied to each experiment are shown in the respective figure legends. We applied both parametric and non-parametric analyses, including Student’s t-test and Mann-Whitney test, according to data distribution. For multiple comparisons, we used one-way ANOVA analysis with Tukey’s or Dunnett’s multiple comparisons.

## Results

### Altered B-Cell Lymphopoiesis in the BM Is a Characteristic of Cd40-KO Recipient Chimeric Mice

**Figure 1:**
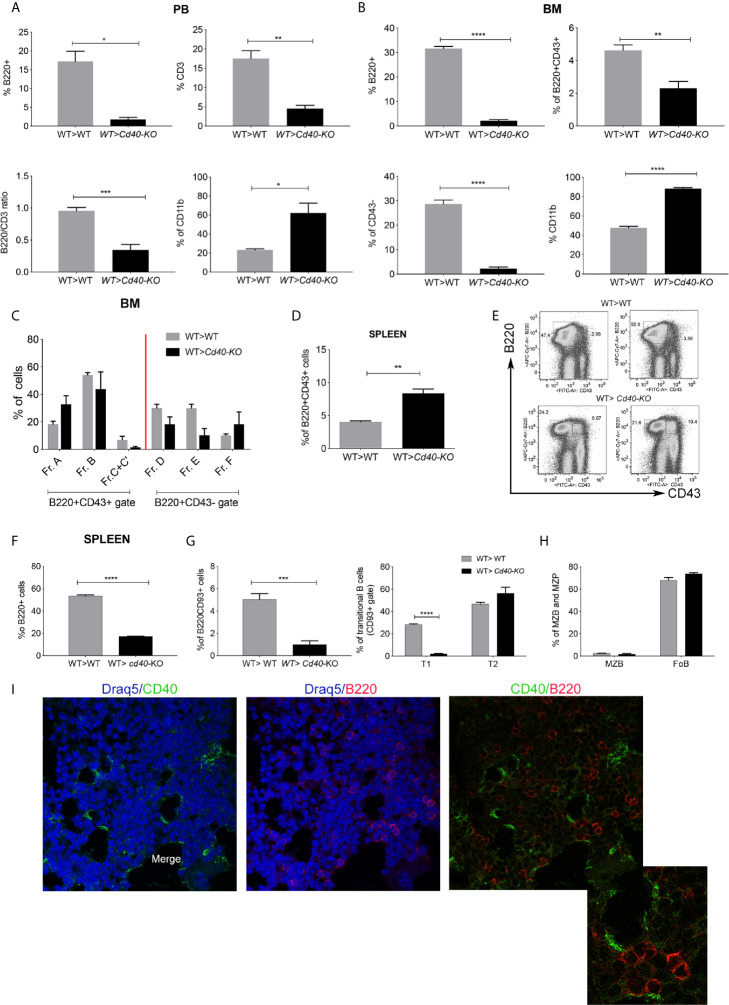
Analysis of B-cell development in WT>WT and WT>*Cd40*-KO BM chimeras. **(A)** Cumulative data from PB FACS analysis showing the frequencies of B220+, CD3+, and CD11b+ cells and also the B220/CD3 ratio in the PB of WT>*Cd40-*KO compared to those of WT>WT BM chimeras. Representative plots and gating strategies are shown in **Supplementary Figure 2A**. **(B)** Cumulative FACS analysis of the BM showing the overall decrease in B220+CD43+ and B220+CD43- B-cell subsets in *Cd40-*KO recipients along with the increase in the frequency of CD11b+ myeloid cells **(C)** Cumulative data showing the frequency of pre-pro-B and early pro-B precursors (A and B fractions) and of late pro-B and large pre-B precursors (C’-C) in chimeric mice. **(D)** Cumulative data showing the frequency of B220+CD43+ B-cell precursors in the spleens of *Cd40-*KO recipient mice. **(E)** Representative dot plots showing that B220+CD43+ cells are nearly absent in the spleens of WT but not *Cd40-*KO recipients. **(F)** Frequency of B220+ B-cells is lower in the spleen of chimeric mice than it is in WT mice. **(G)** Frequency of B220+CD93+ and B220+CD93- B-cells in the spleens of chimeric mice. Splenic immature B220+CD93+ B-cell are divided into transitional T1, T2, and T3 cells based on expression of CD23 and IgM (T1 = IgM + CD23-, T2 = IgM + CD23+, T3 = IgM^low^CD23+). **(H)** Frequency of MZB and FoB within the gate of B220+CD93- cells is also highlighted. The relative gating strategy is shown in **Supplementary Figure 2**. For all panels, the data are derived from 5 mice/group and are representative of one experiment out of 3 performed. Statistical analysis: Student’s *t* test (*p <0.05, **p < 0.005, ***p < 0.001, ****p < 0.0001). **(I)** Immunofluorescence analysis showing CD40+ elements in green and B220+ B-cells in red.

To study the regulatory role of CD40 produced by radio-resistant cells *in vivo*, we performed bone marrow transplant (BMT) experiments where recipient mice that were either WT or *Cd40-*KO were lethally irradiated and then transplanted with HSCs from congenic WT donors. Twenty-one days post-BMT, FACS analyses of peripheral blood (PB), BM, and the spleens of the recipient mice were performed. Compared to WT, *Cd40-*KO recipients exhibited a marked decrease in the frequency of PB B-cells (B220+) and, to a lesser extent, T cells (CD3+), and they also exhibited an increased frequency of myeloid cells (CD11b+)(**Figure 1A**, Additional File 1: **Supplementary Figure 1A**). In agreement with the PB analysis, we observed a reduction in lymphoid cells in the BM of *Cd40-*KO recipients despite normal myelopoietic (CD11b cell) development (**Figure 1B**). In particular, B-cell development was largely defective, as indicated by the reduced B220+CD43+ and almost absent B220+CD43- fractions (**Figure 1B**; Additional File 1: **Supplementary Figure 1B**). B-cell development was arrested at the A and B fractions that correspond to the pre-pro-B and early pro-B phases, respectively, with reduced development of the fraction of C’-C precursors (late pro-B and large pre-B) (**Figure 1C**; Additional File 1: **Supplementary Figure 1B**) (18). Interestingly, in the spleens of *Cd40-*KO recipients, we noted an increased frequency of B220+CD43+ B-cell precursors that developed into mature B-cells of different types, thus giving rise to extramedullary B lymphopoiesis (**Figures 1D, E**). However, the overall frequency of B220+ cells was significantly reduced in the spleens of *Cd40*-KO recipients (**Figure 1F**), where a significant impairment in the CD93+ immature fraction was observed (particularly the transitional T1 cells, **Figure 1G**). Nevertheless, the presence of mature marginal zone B cells and follicular B cells (**Figure 1H**; Additional File 1: **Supplementary Figure 1C**) suggested a compensatory role of splenic B-cell lymphopoiesis in response to dysfunctional BM B-lymphopoiesis. Notably, the defective B-cell lymphopoiesis in the *Cd40-*KO recipient chimeras was not due to a systemic anti-CD40 response, as donor CD40+ B cells were still present in the spleen (Additional File 1: **Supplementary Figure 2A**). These data in combination with the normal B-cell lymphopoiesis of the reverse *Cd40-KO*>WT chimeras (Additional File 1: **Supplementary Figure 2B**) suggest that CD40 expression in radio-resistant cells is necessary to allow for proper BM B-cell development when CD40-competent HSCs are infused. **Figure 1I** shows the close relationship between CD40+ and B220+ cells that supports their functional link in the BM.

### Lethal Irradiation Induces CD40 Expression in BM Radio-Resistant Cells

**Figure 2:**
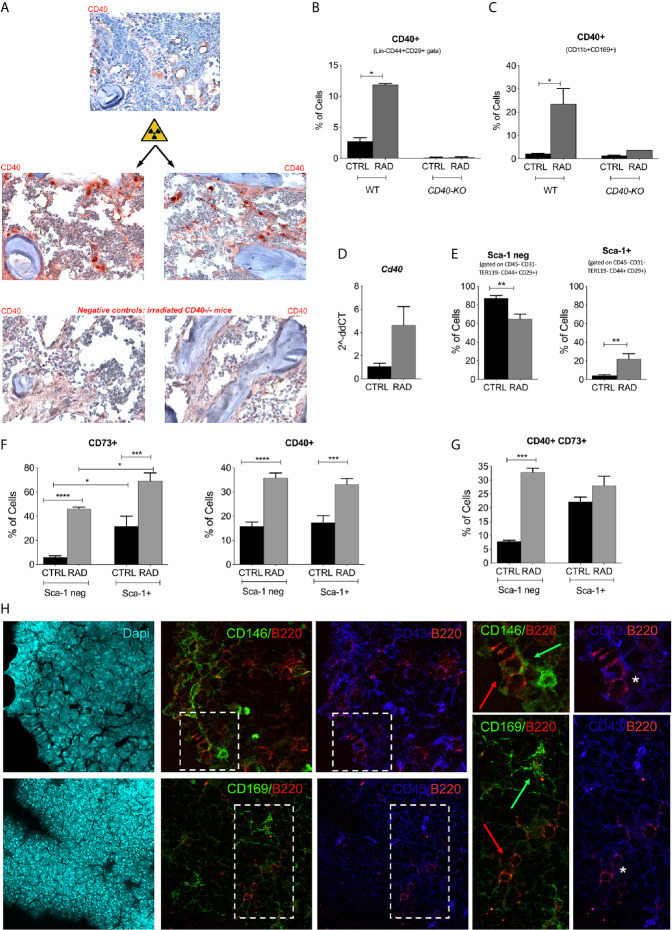
Expression of CD40 in the BM microenvironment. **(A)** Representative image of CD40 IHC staining in the femurs and tibias of WT mice harvested 7 days post-irradiation. **(B)** FACS analysis showing the up-modulation of CD40 on BM-MSC isolated from irradiated (RAD) and non-irradiated (CTRL) BALB/c (CTRL n=5; RAD n=4) and *Cd40-KO* mice (CTRL n=5; RAD n=2). *p < 0.05, Mann-Whitney test. **(C)** FACS analysis showing the up-modulation of CD40 on CD169+ cells isolated from irradiated (RAD) and non-irradiated (CTRL) BALB/c (CTRL n=5; RAD n=4) and *Cd40-KO* mice (CTRL n=5; RAD n=2) *p < 0.05, Mann-Whitney test. **(D)** RT-PCR analysis showing the expression of CD40 on primary BM-MSCs (not expanded *in vitro*) purified from BM at days 4 and 7 post-radiation and compared to basal expression. *p < 0.05, Mann-Whitney test (n=5/group). **(E)** Cumulative FACS analysis showing the frequency of Sca-1+ and Sca-1- BM-MSCs (CD29+CD44+CD45-Ter119-CD31-CD117-CD34- gate) in irradiated mice (**p < 0.005; Student t test; n=8 for controls and n=10 for irradiated mice). **(F)** Cumulative FACS analysis showing the frequency of CD73+ and CD40+ BM-MSCs within the Sca-1+ and Sca-1- gate (n=8 for controls and n=10 for irradiated mice). *p < 0.05, **p < 0.005, ***p < 0.001, ****p < 0.0001, Tukey’s multiple comparisons using a one-way ANOVA. **(G)** Cumulative FACS analysis showing the frequency of CD40+CD73-, CD40+CD73+, and CD40-CD73+ BM-MSCs in irradiated (RAD) *vs* non-irradiated mice (CTRL). n=8 for controls and n=10 for irradiated mice; **p < 0.005, ***p < 0.001, ****p < 0.0001, Tukey’s multiple comparisons using a one-way ANOVA. **(H)** Triple marker confocal microscopy analysis showing CD146+ (green, panels above) or CD169+ (panels below) elements in B220+ (red) and CD43+ (blue) cells. Arrows in the two enlargements indicate the spatial localization of B220+ (red arrow) and CD146+ (green arrow panel above) or CD169+ (green arrow panel below) cells. CD43+B220+ double positive cells (asterisk) are B cell precursors. The analysis indicated that B220+ (in part CD43+) exhibits preferential contact with CD146 but not CD169+ cells.

Next, we evaluated the cellular source of CD40 in the radioresistant compartment of the BM. Lethally irradiated BALB/c mice were sacrificed 7 days post-irradiation for IHC and FACS analysis (20). IHC analysis revealed a strong upregulation of CD40 in the meshwork of radio-resistant cells compared to that in non-irradiated mice (**Figure 2A**). FACS analysis of BM cells demonstrated radiation-induced expansion of CD40+ cells that were characterized as MSCs (**Figure 2B**) or resident CD169+ macrophages (**Figure 2C**). Semi-quantitative PCR analysis confirmed the strong upregulation of CD40 on *ex vivo* purified BM-MSCs (**Figure 2D** and Additional File 1: **Supplementary Figure 3**). To better define the radio-resistant population of BM-MSCs expressing CD40, we evaluated the expression of CD73 markers that characterize radio-resistant MSCs (21) along with known surface MSC markers (CD44, CD29, and Sca-1) and the negative presence of lineage markers CD45, Ter119, CD34, CD117, and CD31. Within the gate specific for CD29+CD44+CD117-Lin- cells, lethal irradiation increased the frequency of Sca-1+ cells while reducing the Sca-1 negative fraction (**Figure 2E**); however, this fraction remained the most representative population of radio-resistant BM-MSCs. In both populations, the fraction of CD73+ and CD40+ cells increased upon irradiation (**Figure 2F**). We next evaluated the frequency of BM-MSCs co-expressing CD40 and CD73, and we observed that irradiation of these cells increased the levels of CD40+CD73+ BM-MSCs, particularly in the Sca-1 negative fraction (**Figure 2G**). The representative gating strategy is presented in **Supplementary Figure 4** (Additional file 1). These data suggest that CD40 and CD73 could be specifically induced and were associated with the radio-resistant compartment of BM-MSCs that could be either Sca-1+ or Sca-1 negative.

The strong impact of *Cd40*-deficiency on B cell precursors suggests that B220+CD43+ precursors are in contact with MSCs or CD169+ macrophages. To further examine this, we performed confocal microscopy analysis for B220, CD43, CD146 (identifying MSC), CD40, and CD169. IF analysis revealed that B220+ CD43+ B cell precursors were in close contact with CD146+ (**Figure 2H**) but not with CD169+ cells (**Figure 2H**). These data support the preferential role of MSC-derived CD40 in the regulation of B-lymphopoiesis. In agreement with this, DTR-mediated deletion of CD169 in the BM altered the development of erythroid lineage cells (22) but not of B cells. Accordingly, *Cd40*-KO and WT recipients exhibited comparable staining for Ter119+ erythroid cells (not shown).

### Radiation-Induced Pro-Inflammatory Cytokines Are Responsible for CD40 Induction in BM-MSCs

**Figure 3:**
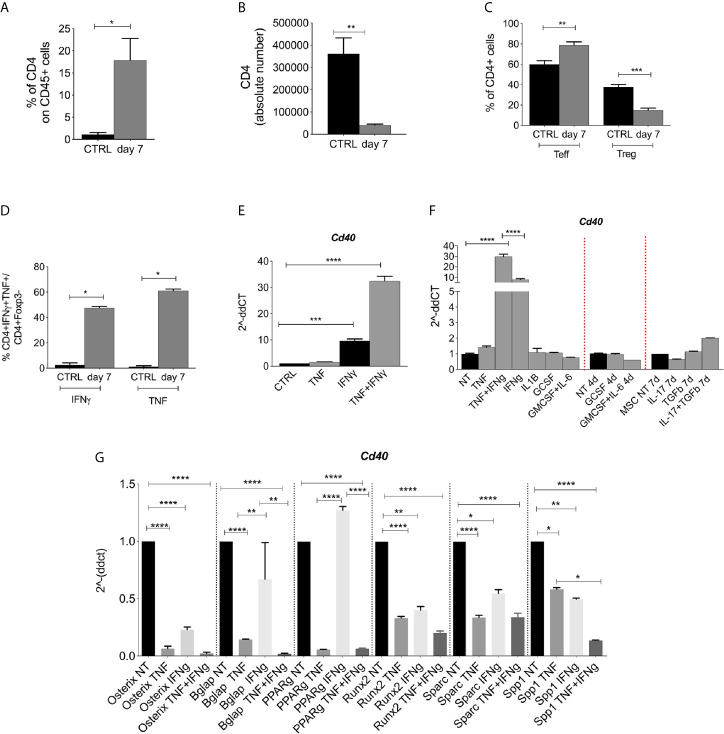
CD40 expression is induced by pro-inflammatory cytokines. **(A)** FACS analysis showing the frequency of CD4+ within the CD45 cells in lethally irradiated (not reconstituted) or naive mice (n = 4 per group). *p < 0.05, Mann-Whitney test. **(B)** Absolute number of CD4+ cells within the BM of irradiated (not reconstituted) or naive mice. (n = 4 per group, *p < 0.05, Mann-Whitney test). **(C)** Frequency of Teffs (CD25-Foxp3−) and Tregs (CD25+Foxp3+) in irradiated mice (CD4 gate) (n = 4 per group). **p < 0.005, ***p < 0.001, compared according to unpaired *t* test. **(D)** FACS analysis showing the frequency of Teffs producing IFNγ and TNF (CD4+Foxp3− gate) (n = 4 per group). **p < 0.005, ***p < 0.001, compared according to unpaired *t* test. **(E)** Semi-quantitative qPCR analysis showing *Cd40* expression on BM-MSCs at 24 h post TNF and IFNg stimulation. ***p < 0.001, ****p <0.0001. (n=3/group, One-way ANOVA, Tukey’s multiple comparison). **(F)** RT-PCR analysis for CD40 expression in BM-MSCs (*ex vivo* isolated and *in vitro* expanded) treated *in vitro* with IL-1b, G-CSF, GM-CSF+IL-6, IL-17, and TGF-b. (****p <0.0001, One-way ANOVA, Tukey’s multiple comparison). **(G)** TNF and IFNg stimulation of BM-MSC alters the differentiation program toward osteo-and adipo- lineages. RT-PCR analyses showed that after 24 h, the treatment with 10 ng/ml IFNg and 50 ng/ml TNFa decreased the expression of osteoblast and adipocytes differentiation markers on BM-MSC compared to levels on untreated cells. The combination of TNF and IFNg was additive in decreasing the expression of *Spp1* (*p < 0.05, **p < 0.005, ****p < 0.0001, One-way ANOVA, Tukey’s multiple comparison).

We then analyzed the radioresistant T-cell compartment that comprises both Teffs (CD4+Foxp3−) and Tregs (CD4+Foxp3+) that are expected to be relevant for the release of cytokines involved in the regulation of CD40 in nearby cells. Compared to the non-irradiated controls, irradiated mice possessed an overall increased frequency of CD4+ T-cells (**Figure 3A**) (CD45+ gate), despite a reduction in absolute T cells (**Figure 3B**). Among radio-resistant CD4+ T-cells, the frequency of Teffs increased at the expense of Tregs (**Figure 3C**). Moreover, Teff was activated and released TNF and IFNγ (**Figure 3D**).

When tested *in vitro*, TNF-and IFN−γ treatments induced CD40 expression in BM-MSCs (**Figure 3E**) more strongly than did other stimuli that are known to be released in the radiation-conditioned BM (i.e., G-CSF, GM-CSF+IL-6, and IL-17) (23) or molecules that are conventionally used to stimulate mesenchymal cells (i.e., TGFβ) (**Figure 3F**). We next tested if the gain of immune regulatory features in BM-MSCs occurred at the expense of their differentiation program toward the osteogenic and adipogenic lineages. RT-PCR analyses were performed on TNF-and IFN -stimulated MSCs. Osteoblast and adipocyte differentiation markers such as osterix, osteonectin, osteopontin, bglap, and PPARg were all downregulated by TNF, and osterix, Runx2, Sparc, and Spp1 but not Bglap and PPARg were downregulated by IFNg (**Figure 3G**). The combination of TNF and IFN was additive in decreasing the expression of *Spp1* (**Figure 3G**). The contrasting, increased expression of CD40 in the same stimulated MSC indicates an inverse relationship between the expression of immune and differentiation programs.

### CD40-Deficiency in the BM Stromal Compartment Creates a Lymphopoietic Niche That Is Unfit for Responding to Stress Condition

Data from the literature indicate a close relationship between B-cell development and regulatory T cells in the BM niche (3). Therefore, we tested if the lack of CD40 expression could affect Tregs.

**Figure 4:**
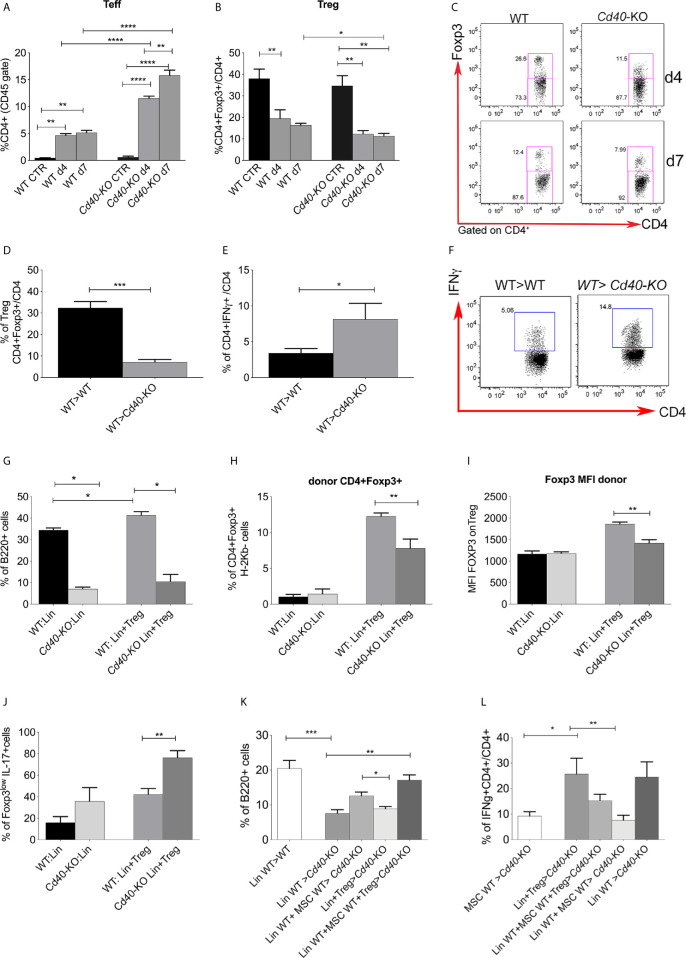
Characterization of T-cell status in the *Cd40*-KO recipient BM chimeras. **(A)** Frequency of Teffs (CD25-Foxp3−) and **(B)** Tregs (CD25+Foxp3+) in WT and *Cd40*-KO irradiated mice (CD4 gate) compared to that in naive control mice (n = 4 per group). **p < 0.005, ***p < 0.001, ****p < 0.0001, Student *t* test. **(C)** Representative dot plot showing Tregs in wt and *Cd40*-KO mice upon irradiation **(D)** Frequency of Tregs in the BM of WT>WT and WT>*Cd40-*KO BM chimeras (n = 4 per group, ***p < 0.001, Student *t* test). **(E)** Cumulative data and **(F)** representative dot gating strategy showing the production of IFNγ by CD4+Foxp3− Teffs in the BM of WT>WT and WT>*Cd40-*KO BM chimeras (n = 4 per group, *p < 0.05, compared according to Student’s *t* test, one representative experiment out of two performed). **(G)** Frequency of B220+ cells in the WT>WT and WT>*Cd40-*KO BM chimeras after lethally irradiating CxB6F1 WT or *Cd40-*KO mice with lin- cells and spleen-derived Tregs from donor BALB/c mice (n = 5 per group). *p < 0.05, compared according to Student’s *t* test. **(H)** Frequency of donor (H-2Kd^+^Kb^-^) CD4+Foxp3+ Tregs in the WT>WT and WT>*Cd40-*KO BM chimeras reconstituted with lin- and Tregs (n = 5 per group). *p < 0.05, compared according to Student’s *t* test. **(I)** Cumulative data showing the MFI of Foxp3 on Treg cells. **(J)** IL-17 production by Foxp3^low^Tregs in *Cd40-*KO recipients (n = 5 per group). **p < 0.005, compared according to Student’s t test. **(K)** Frequency of B-cells in the BM of *Cd40-KO* and *WT* mice receiving Lin- cells co-infused with Treg and MSC from WT donors (n= 4/group, p < 0.05; **p < 0.005, One-way ANOVA, Dunnett’s multiple comparison test). **(L)** Frequency of IFNγ−producing CD4+ cells in BM transplanted *Cd40-KO* and *WT* mice receiving Lin- cells co-infused with Treg and MSC from WT donors (n= 4/group, p < 0.05; **p < 0.005, One-way ANOVA, Dunnett’s multiple comparison test).

The above demonstration of Treg reduction in favor of Teff expansion upon lethal irradiation in WT mice was more pronounced in *Cd40*-KO mice (**Figures 4A–C**). Interestingly, this effect was restricted to the BM and did not occur in the spleen, where the frequency of Teff and Treg was comparable in both WT and *Cd40*-KO mice under the same irradiation conditions (Additional File 1: **Supplementary Figure 5A**).

Next, we analyzed BM T-cell status in WT> *Cd40-*KO versus WT>WT chimeras at 4 weeks after BMT. Tregs were reduced in the BM of *Cd40-*KO recipient chimeras in comparison to levels in the WT counterpart (**Figure 4D**). Despite this, IFNγ-producing Teff cells were significantly increased in the *Cd40-*KO recipient chimeras (**Figures 4E, F**). To gain further insight into the behavior of Tregs in the *Cd40-*KO recipients and their relationships with B-cell development, Tregs were co-injected with donor Lin- cells (both Balb/c, H-2^d^) into either F1 WT or *Cd40-*KO recipients (both CxB6, H-2^b/d^) (Additional File 1: **Supplementary Figure 5B**). According to previous studies (3), the addition of Tregs accelerated the recovery of B cells in the BM of WT>WT but not in WT>*Cd40-*KO chimeras (**Figure 4G**). In agreement with this result, we observed a reduced frequency of donor Tregs in *Cd40-*KO recipients compared to that in WT recipients (**Figure 4H**), and this was paralleled by decreased Foxp3 levels (**Figure 4I**) and increased production of IL-17 in the donor CD4^+^Foxp3^low^ population of *Cd40-*KO recipients (**Figure 4J**). This phenotype suggests a possible conversion of Tregs into Th17 cells (24, 25). Having demonstrated that Tregs were unable to correct the defective B-cell development occurring in *Cd40-KO* recipients, we performed a BMT experiment in which *Cd40-KO* and *WT* mice were transplanted with Lin- cells co-infused with Tregs and MSCs from WT donors. *Cd40-KO* mice that received Lin- cells co-infused with MSCs and Tregs exhibited a B-cell frequency that was similar to that of WT mice transplanted with Lin- cells (**Figure 4K**). Notably, FACS analysis of the T-cell compartment revealed a strong reduction in the inflammatory response in *Cd40-KO* mice that received Lin- cells co-infused with MSCs and Tregs (**Figure 4L**).

### The CD40/CD40L Axis Restrains OX4OL Production by BM-MSCs

Given the strong T-cell activation at the expense of Tregs that are likely converted to a Th17 phenotype that was observed in the *Cd40-KO* recipients, we evaluated the expression of OX40L in the BM of chimeric mice. OX40L is a co-stimulatory molecule for T-cells involved in cytokine production, T-cell expansion, survival, and memory development. However, OX40L is also critical for Treg function and differentiation. Indeed, it blocks Treg suppressive activity and inhibits the generation of new Tregs, thus promoting their differentiation into inflammatory Th17 cells (9–11).

**Figure 5:**
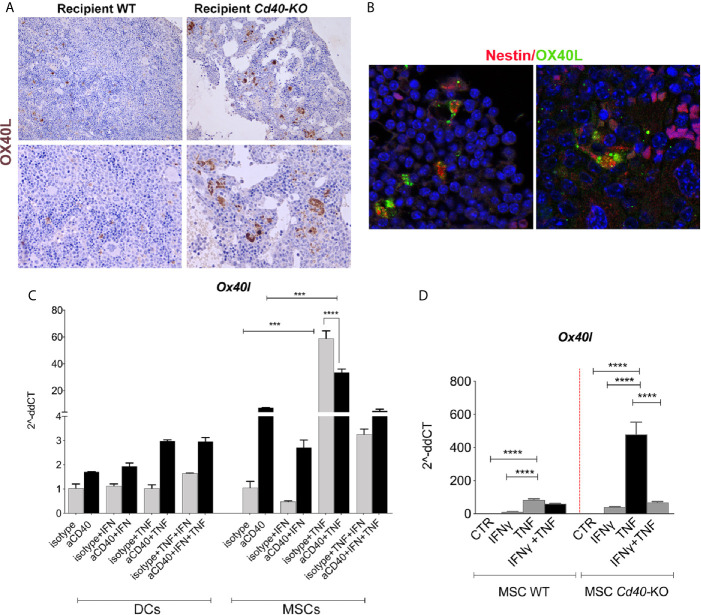
OX40L release is negatively regulated by CD40 triggering. **(A)** Representative images of OX40L staining in BM sections of the WT>WT and WT>*Cd40-*KO BM chimeras. **(B)** IF revealed the co-localization between nestin (BM-MSC marker, red) and OX40L (green). **(C)** *ox40l* expression in Dendritic cells (DCs) and BM-MSCs stimulated with IFN-γ 10 ng/ml, TNF-α 50 ng/ml, and aCD40 mAb or with isotype control (5 μg/ml) for 24 h (n = 3/group, one-way ANOVA, Tukey’s mupltiple comparison). **(D)** Quantitative RT-PCR analysis for *Ox40l* in WT and *Cd40-*KO BM-MSCs treated *in vitro* with IFN-γ, TNF-α, or their combination (***p < 0.001, ****p < 0.0001, ANOVA, Tukey’s multiple comparison).

IHC analysis performed on BM biopsies to compare the different mouse chimeras demonstrated an overall increase in OX40L expression in *Cd40-*KO recipient mice (**Figure 5A**), and confocal microscopy analysis revealed OX40L co-staining with the MSC marker nestin (**Figure 5B** **)**.

Next, we evaluated the mechanisms driving OX40L expression in BM-MSCs. The regulation of OX40L expression in DCs and B cells is known to strictly depend upon CD40 triggering (26). Therefore, we stimulated BM-derived DCs and BM-MSCs with the CD40-agonist mAb CD40 with or without the pro-inflammatory cytokines TNF or IFN. According to the literature, OX40L was induced in DCs solely by CD40 triggering and not by direct exposure to TNF (**Figure 5C**). Conversely, in BM-MSCs, TNF induced OX40L expression that was independent of CD40 triggering (**Figure 5C**). Most notably, the effect of TNF was reduced by concomitant CD40 triggering, thus indicating a novel opposite regulation exerted by CD40 that resulted in the triggering of OX40L expression in BM-MSCs and DCs. Notably, IFNγ was ineffective in inducing OX40L in both BM-MSCs and DCs, thus suggesting a major role of TNF in OX40L induction.

In agreement with this, RT-PCR analysis of *ex vivo* stimulated BM-MSCs revealed that *Cd40-*KO BM-MSCs possessed higher OX40L levels than did their WT counterparts according to the requirement of CD40 triggering to dampen OX40L production (**Figure 5D**).

### BM From aGVHD Patients Lacks Regulatory CD40+ MSC

To provide translational relevance to our findings and to provide a context in humans where CD40+ BM-MSC could be deregulated, we analyzed BM biopsies from patients that underwent HSCT and variably developed aGVHD (Additional File 1: **Supplementary Table 1**). BM manifestations of this disease include a disruption of BM mesenchymal niches by activated T-cells, an event that may affect B-cell lymphopoietic functions (27). Indeed, aGVHD patients exhibit a delayed B-cell recovery in the periphery (28) and contraction of the Treg pull (29). This is a phenotype that is highly similar to that of *Cd40-KO* recipients.

**Figure 6:**
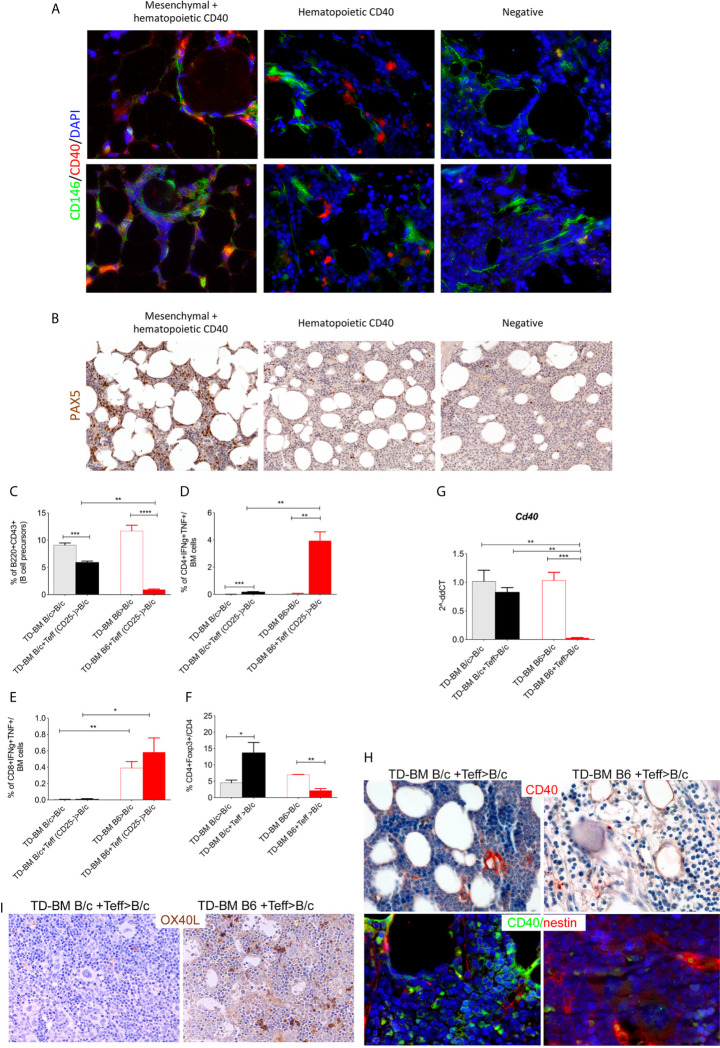
Lack of CD40+ BM-MSCs in BOM from aGVHD patients. **(A)** Representative double-marker immunofluorescence analysis for CD40 (red) and CD146 (green) showing that CD40 expression is shared by CD146+ mesenchymal elements (red arrows) or confined to CD146- hematopoietic cells (white arrows). **(B)** Pax5 IHC showing a variable expression of Pax5, where the highest fractions of Pax5+ cells were observed in cases in which CD40 was expressed also in the mesenchymal cells. Patient characteristics and quantitative IHC data are included in **Supplementary Table 1**. Frequency of B220+CD43+ B-cell precursors **(C)**, CD4+Foxp3− Teffs **(D)**, and CD8+ **(E)** T-cells releasing IFNγ and TNF and the frequency of CD4+Foxp3+ Tregs **(F)** in aGVHD mice (TD-BM B6+Teff> B/c) compared to those characteristics in control animals. Controls were comprised of mice that did not receive MHC-mismatched Teff (TD-BM B6 > B/c) or MHC-matched BM chimeras (TD-BM B/c+/-Teff> B/c) (*p < 0.05, ***p < 0.001, ****p < 0.0001, one-way ANOVA, Tukey’s multiple comparison; n=4/group, one representative experiment out of 3 performed). **(G)** qPCR analysis of *Cd40* expression in BM-MSCs isolated from aGVHD and control mice. **p < 0.005, ***p < 0.001, compared according to ANOVA, Tukey’s multiple comparison. **(H)** Representative CD40 IHC and co-immunofluorescence analysis for CD40 (green) and nestin (red) in BM sections from aGVHD (TD-BM B6 + Teff>BALB/c) compared to levels in controls (TD-BM B/c + Teff>B/c). **(I)** Representative OX40L IHC staining in aGVHD mice (TD-BM B6 + Teff>BALB/c) compared to levels in controls (TD-BM BALB/c + Teff>BALB/c).

Based on this, we speculated if aGVHD could also be associated with a lack of CD40+ MSC regulatory elements in addition to a lack of B-cell precursors (identified through PAX5 staining, an early B-cell transcription factor expressed in the early stages of B-cell lymphopoiesis). To address this, BM biopsies from 12 allo-HSCT patients, seven of which developed aGVHD (grades I-III), were examined for CD40 and PAX5 expression. The patient characteristics are provided in **Supplementary Table 1** (Additional File 1). A blinded analysis of these cases revealed that CD40 was either not expressed or expressed on scattered elements with myeloid morphology or in spindle- or stellate-shaped stromal cells in other cases. The mesenchymal nature of the spindle- or stellate-shaped cells was confirmed through the use of double-marker immunofluorescence analysis for CD40 and CD146 (**Figure 6A**). Pax5 expression ranged from less than 1% to nearly 20% of hematopoietic cells and was positively correlated with CD40 expression in mesenchymal cells. B-cell frequencies were lower in cases exhibiting non-stromal (myeloid) or negative expression of CD40 (**Figure 6B** and **Supplementary Table 1**). The quantification is provided in Additional File 1: **Supplementary Table 1**.

We validated this result using a mouse model of GVHD. We used an experimental model in which major histocompatibility complex (MHC)-mismatched BM cells and lymphocytes isolated from C57BL/6 (H2^b^) donors were transplanted into BALB/c (H2^d^) recipients. T-depleted donor BM (TD-BM) cells (2 106) were co-infused with or without 5 × 10^5^ donor CD4+CD25- (Teff) lymphocytes (30). As a further control, a non-MHC-mismatched BMT was also performed using BALB/c (H2^d^) mice as both donors and recipients (Additional File 1: **Supplementary Figure 6A**). Under allogeneic conditions, aGVHD manifested within 21 days, as confirmed by changes in mouse weight (Additional File 1: **Supplementary Figure 6B**) and histological analysis of the liver, skin, and lung, in accordance with a murine grading system (Additional File 1: **Supplementary Figures 6C–D**) (31). FACS analysis of BM cell suspensions isolated from these mice revealed reduced levels of B cell precursors (B220+CD43+) that were associated with an increased production of IFN -and TNF by CD4 and CD8+ cells (**Figures 6C–E**). Notably, Treg frequency was significantly reduced in the BM of aGVHD mice (**Figure 6F**). Similar changes in the B-cell and Treg populations were also detected in PB samples, where a time-dependent loss of both cell types was observed (data not shown).

We next analyzed CD40 expression in the BM microenvironment during aGVHD. Semi-quantitative RT-PCR analysis revealed loss of CD40 expression in freshly isolated *ex vivo* BM-MSCs from aGVHD but not in control chimeras (**Figure 6G**). CD40 IHC analysis (**Figure 6H**) and co-immunofluorescence analysis for nestin and CD40 (**Figure 6H**) on BM biopsies confirmed the loss of CD40+ mesenchymal cells in aGVHD mice. Notably, the lack of CD40 was coupled with increased OX40L in BM cells (**Figure 6I**), a hallmark of strong T-cell activation.

**Figure 7:**
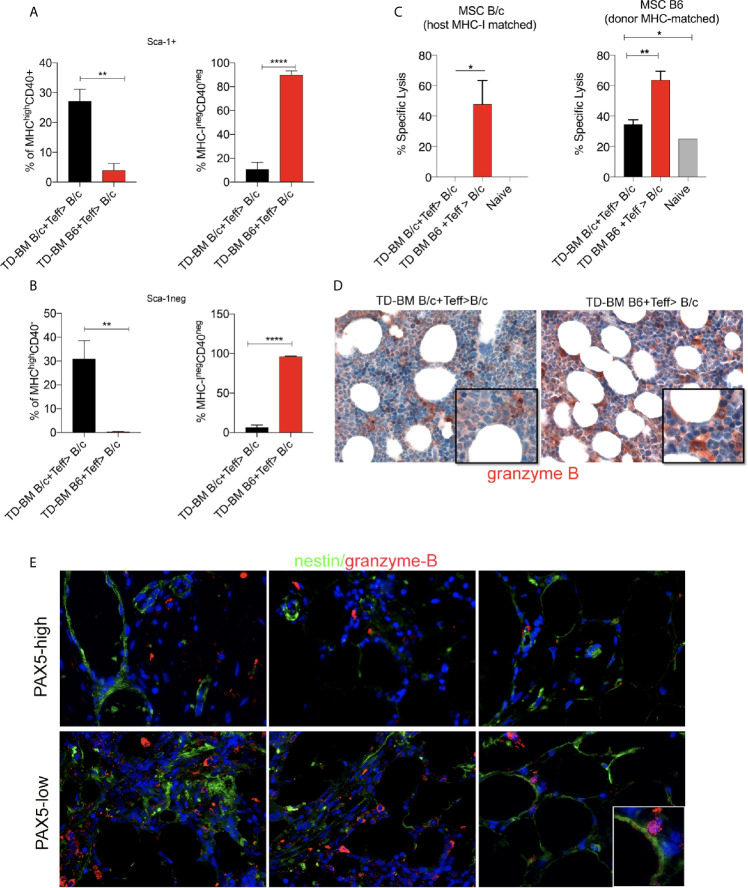
Downregulation of CD40 expression in the BM-MSCs of aGVHD mice. **(A)** Frequency of MHC^high^CD40+, MHC^neg^CD40^neg^ BM-MSCs in the Sca-1+ gate of Lin-CD44+CD29+ BM-MSCs in aGVHD (TD-BM B6 + Teff>BALB/c) *vs* control (TD-BM BALB/c + Teff>BALB/c) mice. The analysis was performed at 14 days post-transplantation. **p < 0.005, ****p < 0.0001, compared according to Student’s *t* test (n=3/group). **(B)** Frequency of MHC^high^CD40+, MHC^neg^CD40^neg^ BM-MSCs in the Sca-1 negative gate of Lin-CD44+CD29+ BM-MSCs in aGVHD (TD-BM B6 + Teff>B/c) *vs* control (TD-BM B/c + Teff>B/c) mice. The analysis was performed at 14 days post-transplantation. **p < 0.005, ****p < 0.0001, compared according to Student’s *t* test. (n = 3/group). **(C)** Cytotoxicity assay performed on MSCs incubated with splenocytes freshly isolated from aGVHD (TD-BM B6 + Teff>B/c) or from controls (TD-BM B/c + Teff>B/c), *p < 0.05, **p < 0.005; **(D)** Representative granzyme B IHC staining of BM biopsies from aGVHD mice (TD-BM B6 + Teff>B/c) compared to that of controls (TD-BM B/c + Teff>B/c) (**p< 0.005, one-way ANOVA, Tukey’s multiple comparison). **(E)** Representative double-marker immunofluorescence analysis for granzyme B (red) and nestin (green) showing the co-localization between granzyme B and nestin+ elements in PAX-5-low patients.

To explain the loss of CD40+ BM-MSCs, we evaluated the expression of MHC-I as a target of allo-recognition of BM-MSCs in lethally irradiated and aGVHD (TD-BM B6+Teff>B/c) *vs.* control (TD-BM B/c+Teff>B/c) mice. Lethal irradiation increased the expression of MHC-I in both Sca-1 negative and-positive fractions of BM-MSCs (Additional File 1: **Supplementary Figure 7**), with both comprising CD40+ elements (**Figure 2F**). The same analysis performed on aGVHD *vs.* control mice at 14 days post-transplantation revealed a dramatic change in the composition of MHC-I+ cells in both the Sca-1+ and Sca-1− fractions (**Figures 7A, B** **)**. In both populations, we observed an almost complete loss of MHC-I^high^CD40+ cells (**Figures 7A, B** **)**, thus leaving intact CD40 negative cells that markedly expressed lower levels of MHC-I (**Figures 7A, B** **)**. These analyses suggest that according to the high MHC-I expression, CD40+ BM-MSCs could be an effective target of donor effector T cells during aGVHD. To test this hypothesis, we evaluated the ability of aGVHD and control transplanted mice to kill CD40+ MSCs from B6 or BALB/c mice using an *ex vivo* cytotoxicity assay. To perform this experiment, MSCs (of both host and donor MHC-I, B/c or B6), were treated with IFN for 24 h to promote the expression of MHC-I and CD40 and then labeled with CFSE and incubated for 4 h with splenocytes from aGVHD and control mice. Cytotoxicity was evaluated using flow cytometry (Additional File 1: **Supplementary Figure 7B**). We observed that GVHD mice, but not controls, efficiently killed host MSCs (**Figure 7C**). Interestingly, splenocytes from aGVHD mice also possessed the ability to lyse donor MHC-matched MSCs. This may indicate the activation of residual radio-resistant host T-cells toward cells of donor origin. In agreement with these data, the IHC analysis revealed a higher expression of granzyme B in the BM of mice developing aGVHD (**Figure 7D**).

In support of the mouse data, BM from GVHD patients revealed that patients with a strong reduction in PAX5+ cells possessed granzyme B+ granules that were localized to residual nestin+ BM-MSCs cells (**Figure 7E**).

## Discussion

The data presented here provide insight into the role of co-stimulatory molecules in harnessing the immune regulatory functions of BM-MSCs and controlling BM T-cell homeostasis.

We revealed that inflammatory stimuli coordinately promote CD40 and OX40L expression on BM-MSCs, ultimately fueling Teffs at the expense of Tregs. However, the induction of CD40 prevents the development of excessive inflammatory conditions through negative regulation of OX40L.

The induction of OX40L in BM-MSCs by TNF represents a strong element of novelty in the physiology of OX40L, and this has been best characterized in DCs, where CD40 triggering is required to induce OX40L. Conversely, in BM-MSCs, we demonstrated that CD40 triggering is required to inhibit the production of OX40L induced by TNF. This fits well with the different needs of DCs in SLO and BM-MSCs in BM to regulate their tissue-specific immune responses that are either stimulatory or regulatory, respectively. Excessive T-cell activation and inflammation negatively impact lymphopoiesis while fostering myelopoiesis (2). This can explain why CD40 is expressed on BM-MSCs only in the case of BM perturbation and not during normal homeostatic conditions. Indeed, lethal irradiation promotes an inflammatory response that is guided by IFN and TNF and is sufficient to induce CD40. Rapid upregulation of CD40 in radio-resistant BM-MSCs is required to sustain Treg functions and to down-modulate inflammatory mediators such as B-cell lymphopoiesis. In support of this, experiments examining Lin- cells that were co-infused with Treg demonstrated a positive effect of Tregs on B-cell recovery in WT but not in *Cd40-KO* recipients. In these mice, the absence of CD40 promoted the conversion of donor Tregs into IL-17-producing Foxp3low cells. This event was associated with defective B-cell development in *Cd40-KO* mice, a condition that in humans can be observed in patients who underwent HSCT and that variably developed aGVHD. Indeed, aGVHD patients exhibit a delayed peripheral B-cell recovery that is associated with increased BM T-cell infiltration oriented towards a Th17 activation state (32). Furthermore, aGVHD patients are characterized by BM Treg defects that are negatively associated with hematopoietic reconstitution (33).

The observed commonalities between certain aspects of GVHD BM manifestations and the phenotype of *Cd40-KO* recipients suggested evaluating CD40+ BM-MSCs in aGVHD patients. By analyzing the BM biopsies from these patients, we demonstrated a loss in CD40+ mesenchymal cells that was paralleled by the loss of PAX5+ B-cell marrow precursor.

By modeling GVHD in mice, we revealed a possible selective elimination of CD40+ BM-MSCs during aGVHD onset, as indicated by their higher MHC-I expression. *In vitro* cytotoxicity experiments demonstrated the allorecognition of CD40^+^MHC^+^ MSCs. Furthermore, in aGVHD biopsies, granzyme-B+ granules were observed to be associated with residual CD146+ and CD40+ BM-MSCs.

The exacerbation of Teff activation may depend upon the residual CD40^neg^MSC network that senses the cytokine storm (particularly TNF, which is a key cytokine in the pathophysiology of aGVHD (34)) and upregulates OX40L expression with a negative impact on Treg function. In agreement with this, mice with aGVHD lacked CD40+ regulatory MSCs but possessed increased OX40L expression on the residual MSC meshwork. The loss of CD40+regulatory BM-MSCs *via* allo-recognition may provide the first indication of impaired Treg activities and initial cytokine storm and Teff activation, all of which in turn sustain further OX40L expression.

OX40L inhibition resulted in positive effects on aGVHD development in both mouse and human non-primate aGVHD models. In this context, Tchaved et al. revealed that a combination of mTOR inhibition and OX40L blockage resulted in reduced damaging Tcell reconstitution but preserved regulatory T-cell activities (35), thus suggesting the double need for blocking Teff and promoting Treg to efficiently inhibit GVHD.

Finally, our study revealed that the gain of regulatory functions along with CD40 upregulation in MSCs is associated with the downregulation of their speciation markers (Bglap, Osterix, and Osteopontin) in favor of immune regulatory features. This can be explained by a more undifferentiated/progenitor subset of BM-MSCs that exerts these functions or by a switch from an architectural/supportive hematopoietic niche function of BM-MSCs to immunoregulatory activity when excessive inflammatory conditions are sensed. Additionally, we described the expression of CD40 in the Sca-1+ population of BM-MSCs that identifies stromal progenitors capable of generating both osteogenic and stromal cells and can provide a supportive environment for hematopoiesis. It has been reported that Sca-1-positive cells exhibit higher colony-forming ability and enhanced proliferation compared to these functions in Sca-1-negative cells (36). This evidence supports the possibility that the subset of BM-MSCs that are primarily endowed with immune regulatory function could be predominantly undifferentiated.

Overall, our study demonstrates that CD40+ BM-MSCs are immune regulatory elements of the bone marrow, and their function results in the inability of the marrow to restrain T-cell activation and restore immune homeostasis that is required to support B-cell development. GVHD is an example of how the loss of these cells results in functional consequences in a human setting.

## Conclusions

BM-MSCs express co-stimulatory molecules that preserve BM T cell homeostasis.

Inflammatory stimuli coordinately promote CD40 and OX40L expression on BM-MSCs to fuel Teff at the expense of Tregs. However, the induction of CD40 prevents the development of excessive inflammatory conditions through negative regulation of OX40L.

## Data Availability Statement

The raw data supporting the conclusions of this article will be made available by the authors, without undue reservation.

## Ethics Statement

Ethical review and approval was not required for the study on human participants in accordance with the local legislation and institutional requirements. The patients/participants provided their written informed consent to participate in this study. The animal study was reviewed and approved by Italian Ministry of Health.

## Author Contributions

SS and MCo designed the research. BB, AG, PP, LB, and MCi performed the experiments. SS, I-KN, KJ, and IA provided human biopsies and analyzed the clinical parameters. CC, CT, and AC analyzed the data. SS, BB, NB, CT, and MCo wrote the manuscript. All authors contributed to the article and approved the submitted version.

## Funding

This work was supported by the Italian Ministry of Health (GR-2013-02355637 to SS) and Associazione Italiana per la Ricerca sul Cancro (Investigator Grant number 22204 to SS).

## Conflict of Interest

The authors declare that the research was conducted in the absence of any commercial or financial relationships that could be construed as a potential conflict of interest.

## Abbreviations

BM, bone marrow; DCs, dendritic cells; FBS, fetal bovine serum; GVHD, graft *vs* host disease; HSC, hematopoietic stem cells; IFN, interferon; IL-, interleukin; MSCs, mesenchymal cells; SLO, secondary lymphoid organs; Teff, effector T cells; TNF, tumor necrosis factor; Treg, regulatory T cells; WT, wild type.
