# Supplementary material for: CD40 Activity on Mesenchymal Cells Negatively Regulates OX40L to Maintain Bone Marrow Immune Homeostasis Under Stress Conditions
Source: Front Immunol. 2021 May 18;12:662048. doi: 10.3389/fimmu.2021.662048 (PMC8168593; doi:10.3389/fimmu.2021.662048)
Supplement: Supplementary file 1 [file DataSheet_1.pdf]

SUPPLEMENTARY METHODS, FIGURES AND TABLES

METHODS

Histology. In GVHD experiments, histological damage was evaluated in the skin, liver, lung and kidney according to a semiquantitative scoring system based on histopathological analysis of routinely-stained haematoxylin and eosin 4-micra-thick tissue sections. In detail, epidermal necrosis, epidermal inflammatory infiltration, and dermal inflammatory infiltration were analysed in the skin; parenchymal inflammatory infiltration, peri-portal necrosis, and centro-lobular necrosis were assessed in the liver; interstitial inflammatory infiltration, epithelial proliferation, and stromal proliferation were scored in the lung parenchyma; glomerular rarefaction, glomerular necrosis, glomerular inflammatory infiltration, and interstitial inflammatory infiltration were evaluated in the kidney. Each variable was scored according to a four-grade semiquantitative score: 0 (absent), 1 (focal/mild), 2 (multifocal/moderate), 3 (diffuse/intense). The final damage score was calculated for each experimental condition by averaging the scores relative to the same tissues.

Lentiviral vector construction, virus production and MSC infection. To construct CXCR4-expressing lentivector, we modified the self-inactivating lentiviral vector pRRLCMVGFPsin-18 (kind gift of Dr. G. Ferrari) by replacing the GFP sequence with mouse CXCR4 cDNA (Genescript). A third-generation packaging system (pMDLg/pRRE and pRSV-REV and pMD2-VSV-G and transfer vectors) was used to produce viral particles. Lentiviral stocks were produced in 293T cells by Ca_3PO_4 co-trasfection of the four plasmids. Twenty-four hours after transfection, the virus containing supernatant was harvested, filtered and purified by ultracentrifugation. The viral titer was calculated by analysis of the expression of CXCR4 by flow cytometry in 293T cells infected with different dilution of virus. A MOI 50 was used to infect MSC.

Real Time PCR. Total RNA was extracted by using the Quick RNA micro prep kit (Zymo Research) and subsequently quantified with NanoDrop 2000c Spectrophotometer (Thermo Scientific). MultiScribe-Reverse Transcriptase kit (Applied Biosystems) was used for the reverse transcription assay and the Real-Time PCR was performed using the Taqman Universal PCR Master Mix (Applied Biosystems) according to the manufacturer instructions. Briefly, the master mix, 20 ng of cDNA and the probes of interest were diluted in a total volume of 20 μL and the Real-Time PCR was performed on 7900HT Fast Real-Time PCR System (Applied Biosystems).

Taqman Probes: *Cd40* (*Mm00441891_m1*), *Tnfsf4* (*ox40l - Mm00437214_m1*), *Bglap (Mm03413826_mH), sp7 (osterix - Mm00504574_m1), pparg (Mm00440940_m1), runx2 (Mm00501584_m1), sparc* (*Mm00486332_m1), spp1 (Mm00436767_m1).*

IHC and immunofluorescence analyses. For IHC, Human and Murine BM samples were fixed in 10% buffered formalin, decalcified using an EDTA-based buffer, and paraffin-embedded. Tissue

sections (4 μm) were deparaffinized and rehydrated. An antigen unmasking technique was performed using Novocastra Epitope Retrieval Solution (pH 9) in a PT Link Dako pre-treatment module at 98°C for 30 min. Subsequently, the sections were brought to room temperature and washed in PBS. After neutralization of the endogenous peroxidases with 3% H2O2 and Fc-blocking by a specific protein block (Novocastra, UK), the samples were incubated overnight at 4°C with primary antibodies listed in Supplementary Table 2. The immunostaining was revealed by either a polymer detection method (Novolink Polymer Detection Systems Novocastra Leica Biosystems Newcastle Ltd Product No: RE7280-K), and following specific secondary antibodies: horseradish peroxidase (HRP)-conjugated donkey anti-rabbit IgG (H+L)(A16035, Invitrogen) or HRP-conjugated goat anti-rat IgG (H+L) (Vector Lab) secondary antibody and either 3-amino-9-ethylcarbazole (AEC) or 3,3'-diaminobenzidine (DAB) substrate-chromogens.

For co-immunofluorescence, BM and spleen sections were pretreated as detailed above for IHC. The primary antibodies used for this analysis are detailed in Supplementary Table 2. Primary antibody binding was amplified and visualized using Alexa Fluor 568-conjugated goat anti-rabbit IgG (H+L) (A11011, Invitrogen), Alexa Fluor 488-conjugated goat anti-rat IgG (H+L) (A11006, Invitrogen), Alexa Fluor 488-conjugated goat-anti-mouse IgG (H+L) (A11001, Invitrogen), or Alexa Fluor 633-conjugated goat anti- rat IgG (H+L) (A21094, Invitrogen). The slides were counterstained with DAPI Nucleic Acid Stain (Invitrogen Molecular Probes). The samples were then analyzed under an Axioscope A1 optical microscope (Zeiss) and microphotographs were collected using an Axiocam 503 color digital camera (Zeiss). Images were analyzed using Zen2 imaging software.

The quantitative analysis of CD40 and PAX5 in BM biopsies from human samples by immunohistochemistry was performed on whole sections scans using a Leica Aperio CS2 slide scanner and by quantifying the staining by two different software tools. The Nulcear Hub tool (Nuclear v9) of the Image Scope software was used to determine the percentage of PAX5-expressing nuclei over the total cellularity, while the Positive Pixel Count v9 tool was adopted to categorize the CD40-stained slides according to a semi-quantitative score ranging from 0 (absent) to 3 (diffuse/intense).

SUPPLEMENTARY FIGURES

Figure S1

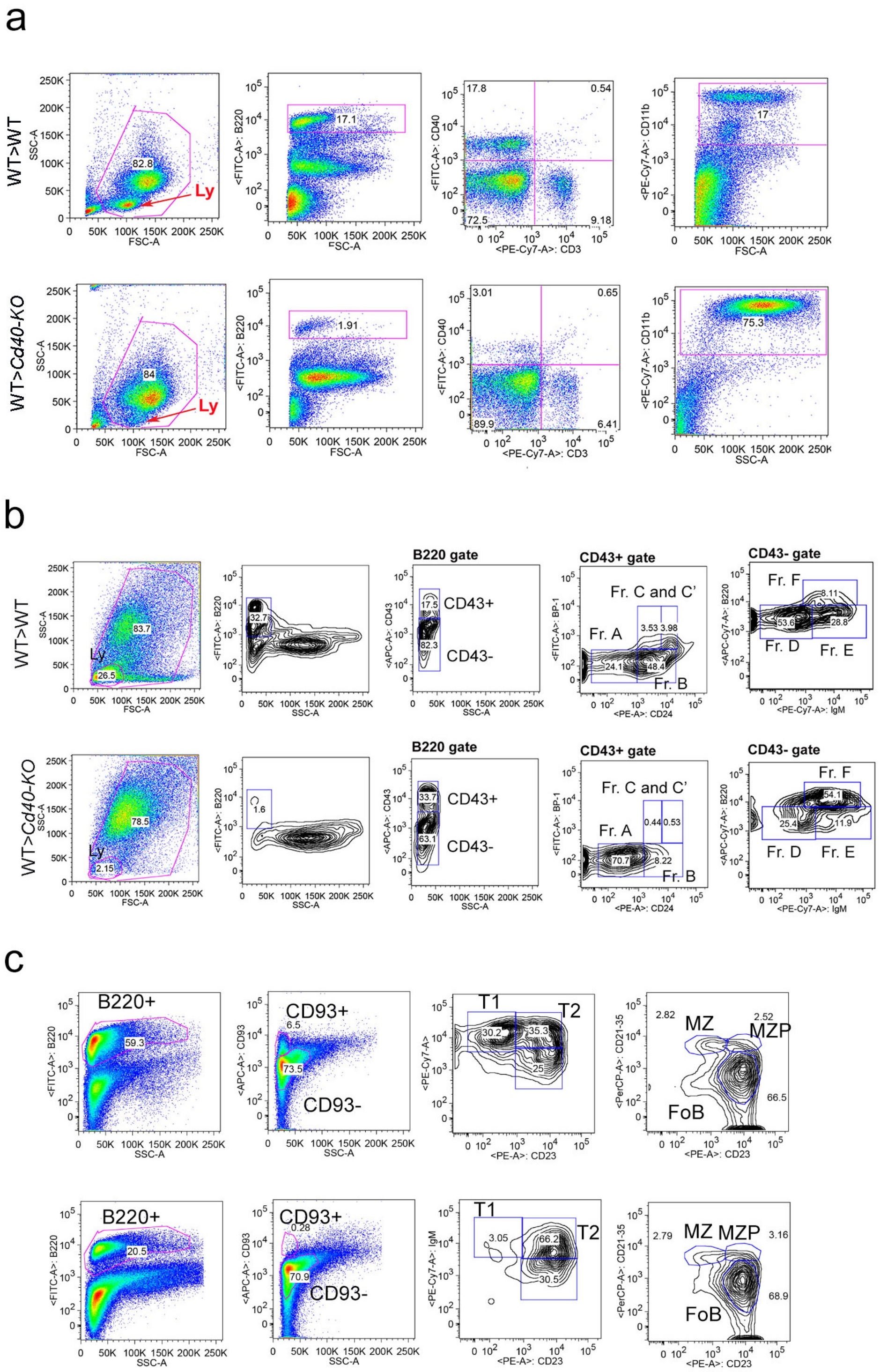

Gating strategy utilized to characterize B-cell development in mouse BM chimeras.

a. Representative gating strategy for PB FACS analysis showing the frequencies of B220+, CD3 and CD11b+ cells in the PB of WT>*Cd40*-KO BM chimeras compared to WT>WT BM chimeras. **b.** Representative dot plots showing the gating strategy used to characterize BM B-cell development in chimeric mice. CD43+ B-cell precursors are identified within the B220+ gate. CD43+ cells are further characterized according to the expression of BP-1 and CD24 that allows to

distinguish among the different pre-pro and pro-B fractions (A, B, C and C'). Within the CD43- population the expression of IgM and B220 identified the fraction D, E and F **c** representative dot plots showing the different B-cell populations maturating in the spleens of WT>WT and WT> *Cd40*-KO BM chimeras. Splenic immature B220+CD93+ B-cell were divided into transitional T1, T2, and T3 cells based on their expression of CD23 and IgM (T1 = IgM + CD23-, T2 = IgM + CD23+, T3 = IgM^{low}CD23+).

Figure S2

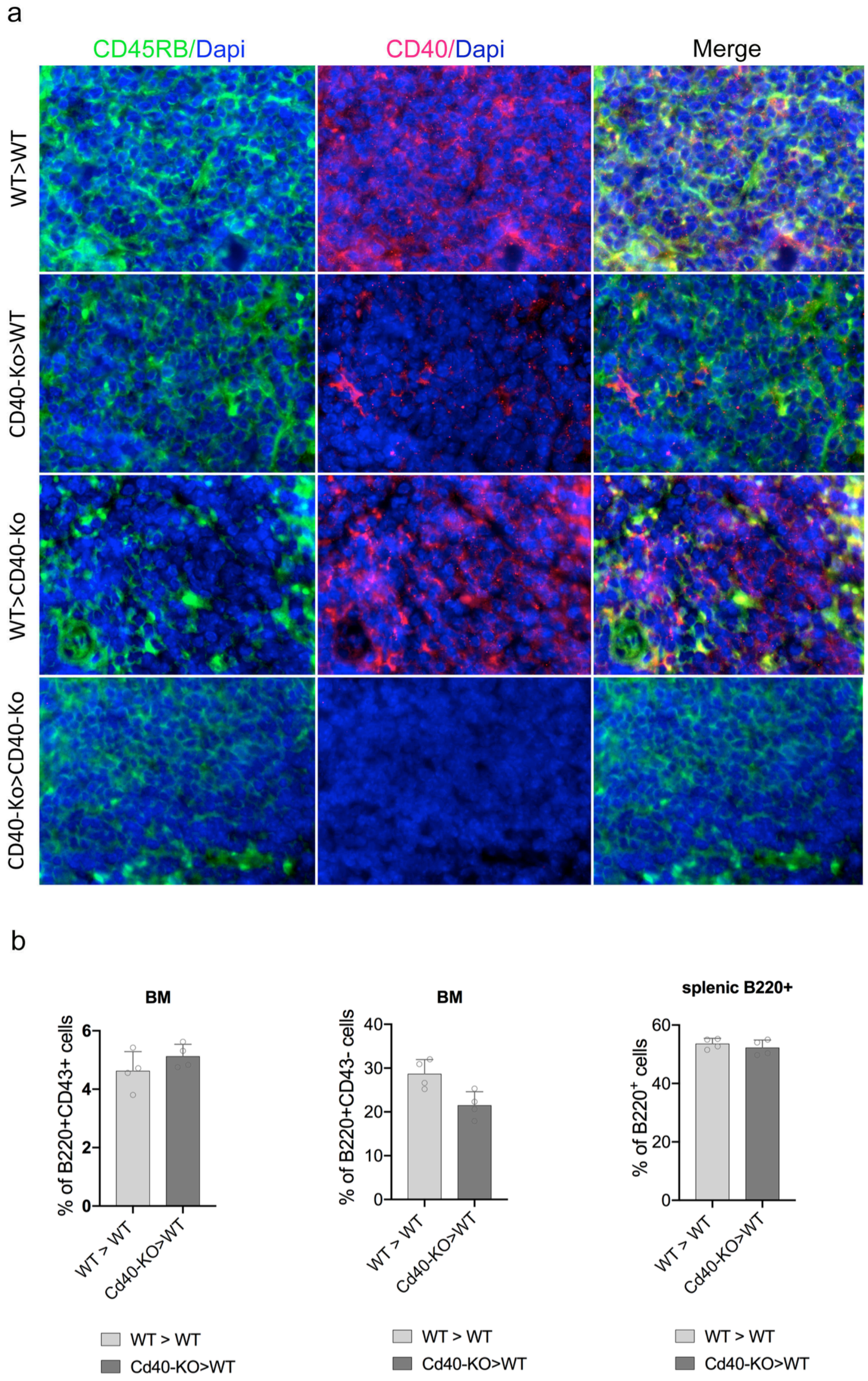

Detection of B220+CD40+ B-cells in Cd40-KO recipient mice. **a.** Representative IF microphotographs of spleen sections from different chimeric mice showing the presence of B220+(green signal) CD40+(red signal) cells in the spleen of *WT>WT* and *WT>Cd40-KO* chimeras. In *Cd40-KO>WT* chimeras CD40 expression is confined to scattered CD45RB B220-negative

stromal elements while *Cd40-KO*>*Cd40-KO* controls have no detectable CD40 expression (Original magnification x400). **b.** Frequency of B220+CD43+ and B220+CD43- cells within the BM and percentage of splenic B220+ cells of *WT*>*WT* and *WT*>*Cd40-KO* chimeras

Figure S3

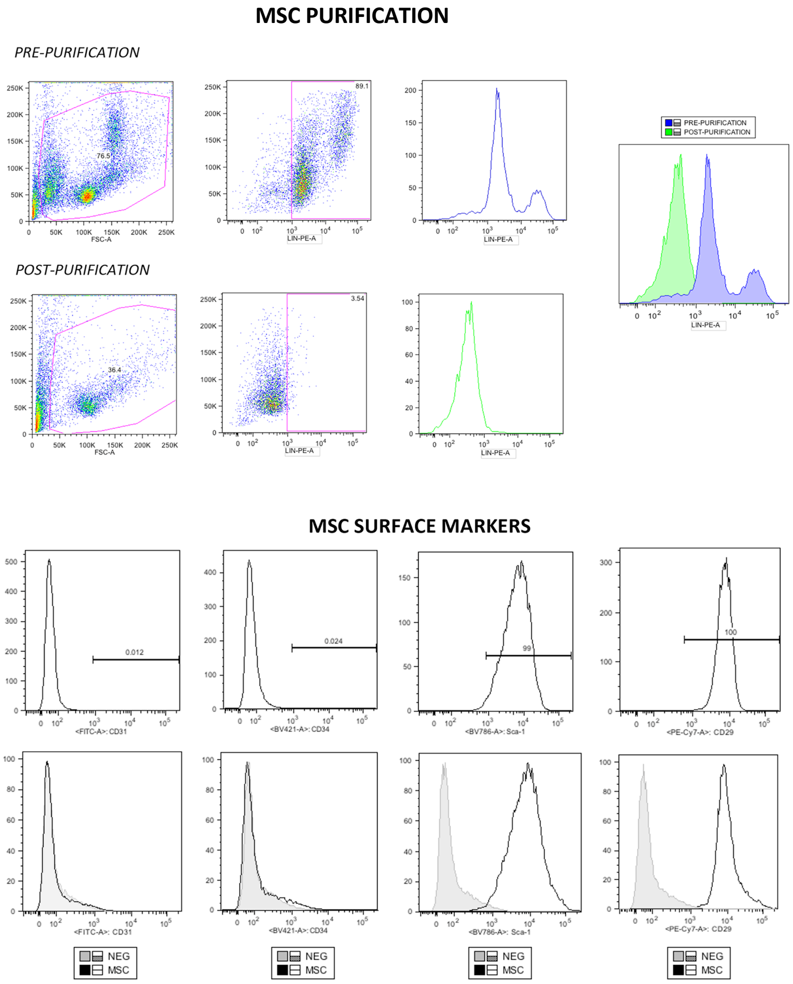

BM-MSC purification and phenotypic characterization. Phenotypic characterization of purified mesenchymal stem cells – Following the enzymatic digestion with collagenase 0,2 mg/mL, cell suspension is filtered by using 70 □m cell strainers and incubated with the Lin antibody cocktail that includes CD45, CD11b, CD11c, CD3, GR-1, F4-80, B220, TER119 PE-conjugated. Then cells where labeled with Anti-PE MicroBeads (Miltenyi Biotech) and magnetically separated. Representative dot plots and the relative histograms of the samples pre- and post-purification are shown MSC phenotype was deeper investigated using CD34, CD31, CD29 and Sca-1 antibodies and the expression of the selected surface markers is shown by representative histograms

Figure S4

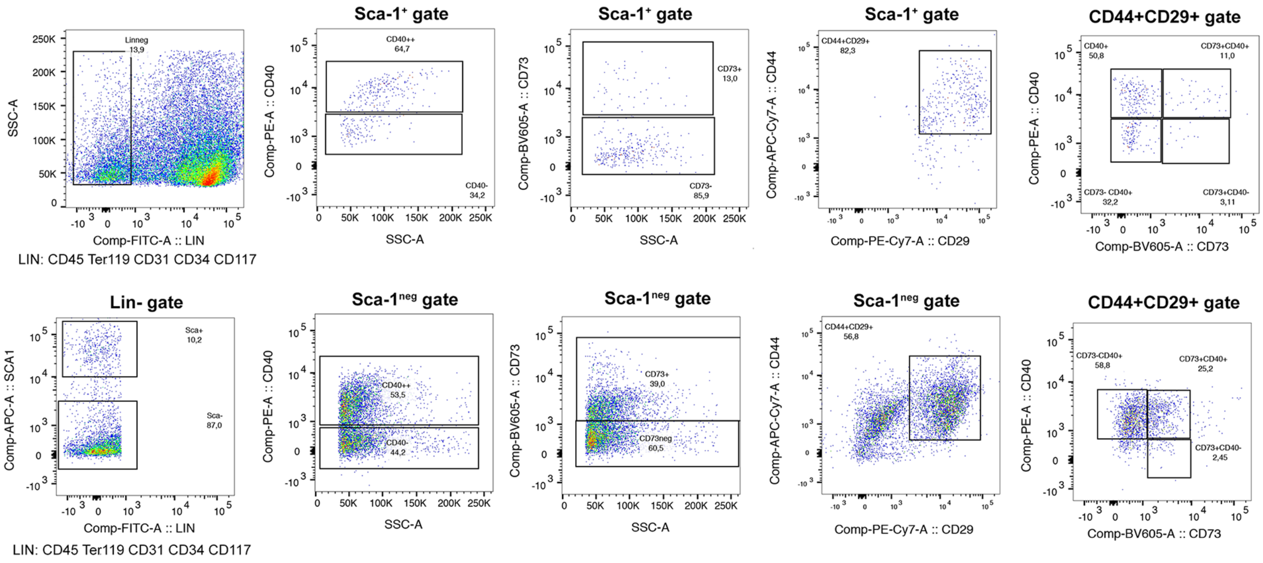

Expression of CD73 and CD40 on Sca-1^{+} and Sca-1^{neg} BM-MSC MSCs. Representative gating strategy for CD73 positive, CD40 positive and CD40/CD73 double positive MSCs is shown.

Figure S5

A

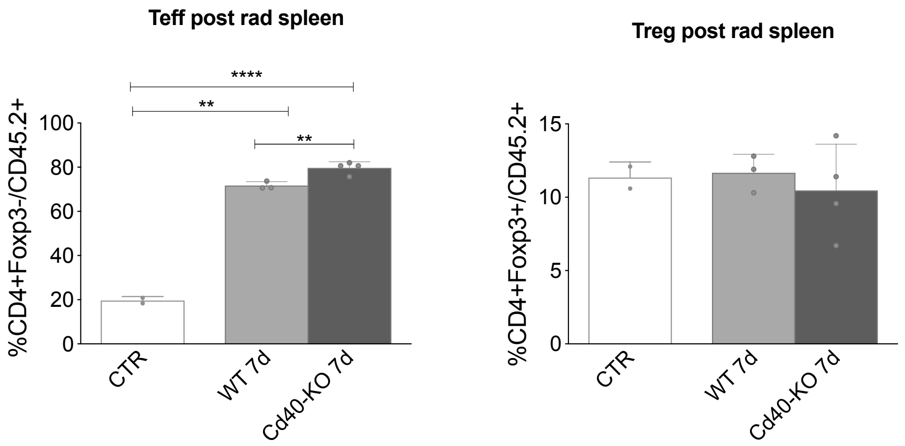

B

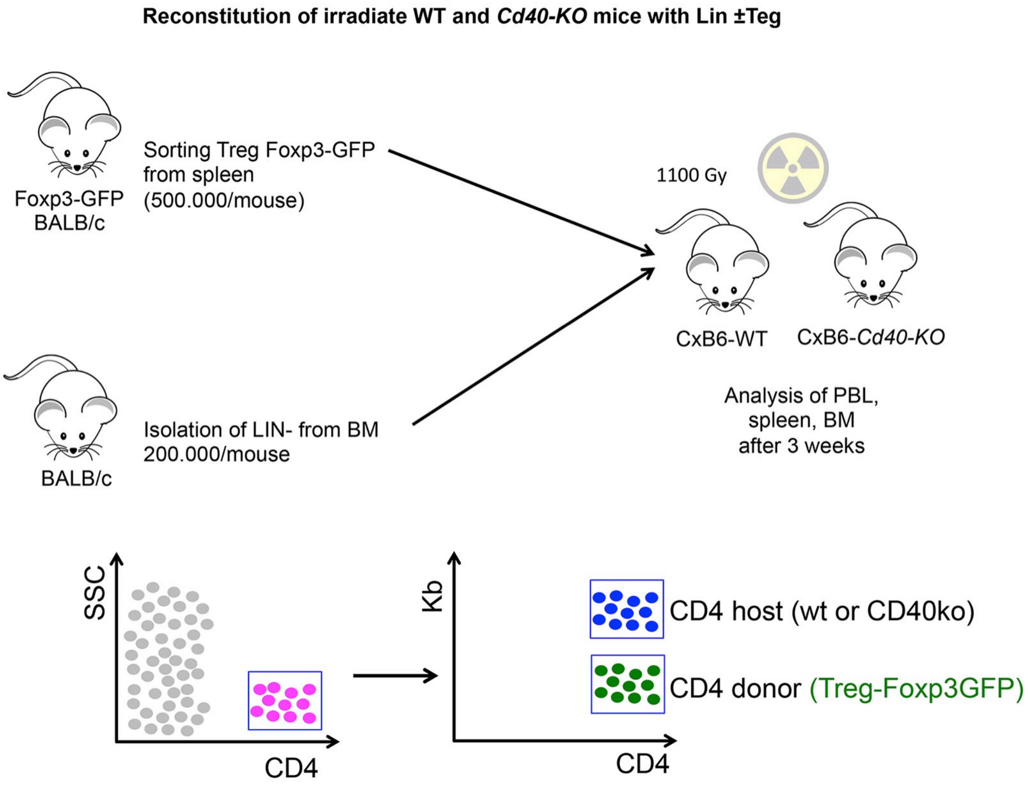

Treg behavior in wt and cd40-KO mice. a. Frequency of Teff and Treg within the spleen of irradiated WT and *Cd40-KO* mice. b. Schematic representation of BMT experiments in which Lin-cell were co-injected with Treg cells into lethally irradiated WT and *Cd40-KO* mice. The use of CxB6 F1 mice (either WT or *Cd40-KO*) as recipients allowed discriminating donor and host Treg, being donor Treg H-2Kb negative.

Figure S6

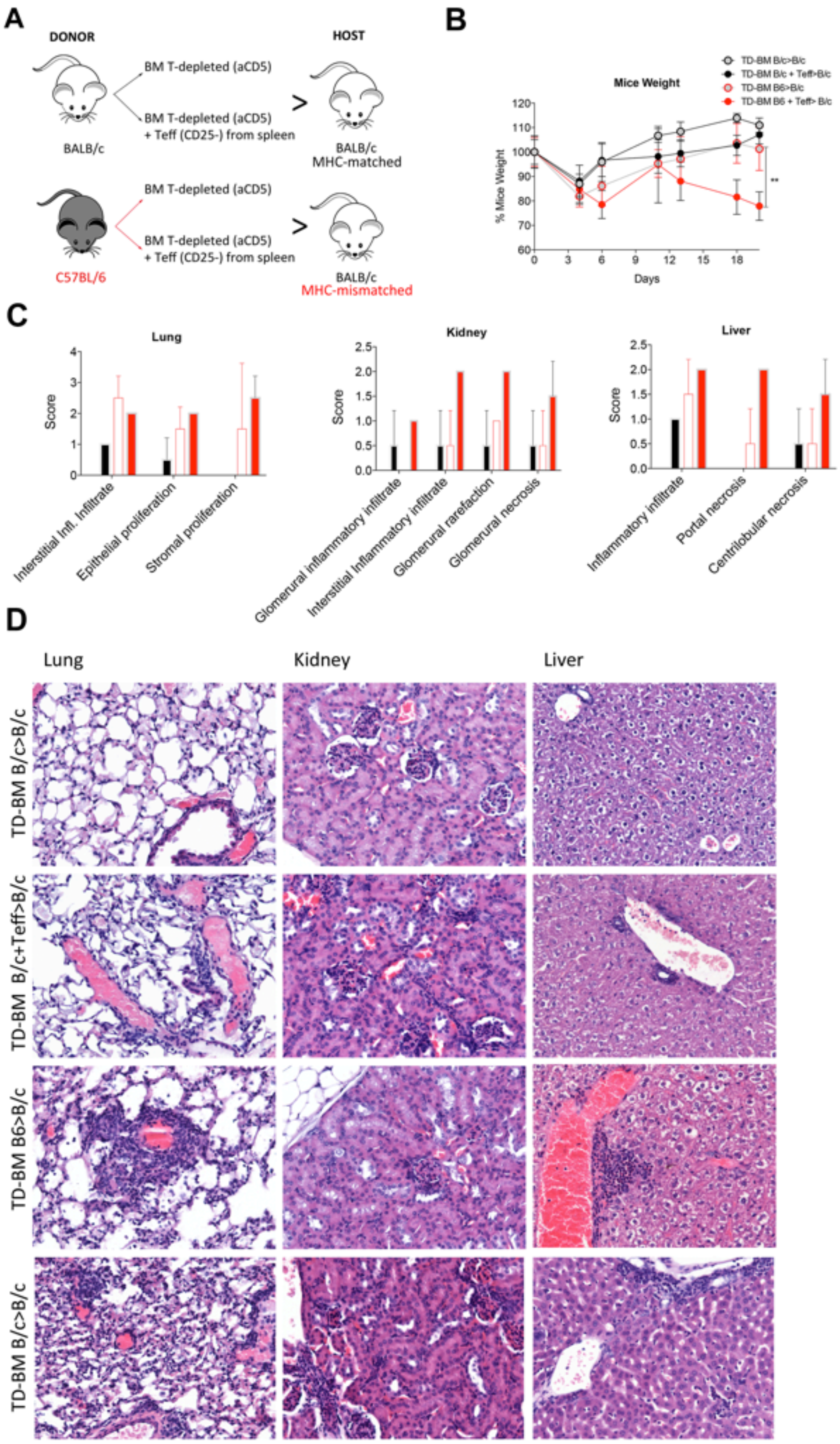

A
DONOR
HOST
BM T-depleted (aCD5)
BM T-depleted (aCD5)
+ Teff (CD25-) from spleen
BALB/c
BALB/c
MHC-matched
BM T-depleted (aCD5)
BM T-depleted (aCD5)
+ Teff (CD25-) from spleen
C57BL/6
BALB/c
MHC-mismatched
B
Mice Weight
TD-BM B/c>B/c
TD-BM B/c + Teff>B/c
TD-BM B6>B/c
TD-BM B6 + Teff> B/c
% Mice Weight
Days
**
C
Lung
Score
Interstitial Infl. Infiltrate
Epithelial proliferation
Stromal proliferation
Kidney
Glomerural inflammatory infiltrate
Interstitial Inflammatory infiltrate
Glomerural rarefaction
Glomerural necrosis
Liver
Inflammatory infiltrate
Portal necrosis
Centrilobular necrosis
D
Lung
Kidney
Liver
TD-BM B/c>B/c
TD-BM B/c+Teff>B/c
TD-BM B6>B/c
TD-BM B/c>B/c

Histological features of aGVHD mouse models. **a.** Schematic representation of the BMT experiment performed to obtain MHC-mismatched (modeling aGVHD) or non-MHC-mismatched BM chimeras. **b** Weight loss in aGVHD at day 21 after BMT in the specified allogeneic-transplanted and control animals (n= 12 per group). **p < 0.005, compared using Student's *t* test. **c**. Histological score for lung, liver, kidney and skin damage in aGVHD mice (TD-BM+Teff B6>B/c) and control groups. **d.** Representative microphotographs detailing the different extent of immune infiltration and associated histological damage in the lung, kidney and liver parenchyma of mice belonging to the four groups. No significant immune cell infiltration is detected in the tissues of TD-BM B/c>B/c mice; slight inflammatory infiltration mainly confined to peri-vascular areas and without signs of parenchymal damage characterizes TD-BM B/c+Teff>B/c mice; Focally severe inflammatory infiltration associated with evidence of parenchymal damage is detected in TD-BM B6c>B/c mice samples; Multifocal or diffuse immune infiltration determines overt disarrangement of the histological architecture in the three organs of TD-BM B6+Teff>B/c mice.

Supplementary Figure 7

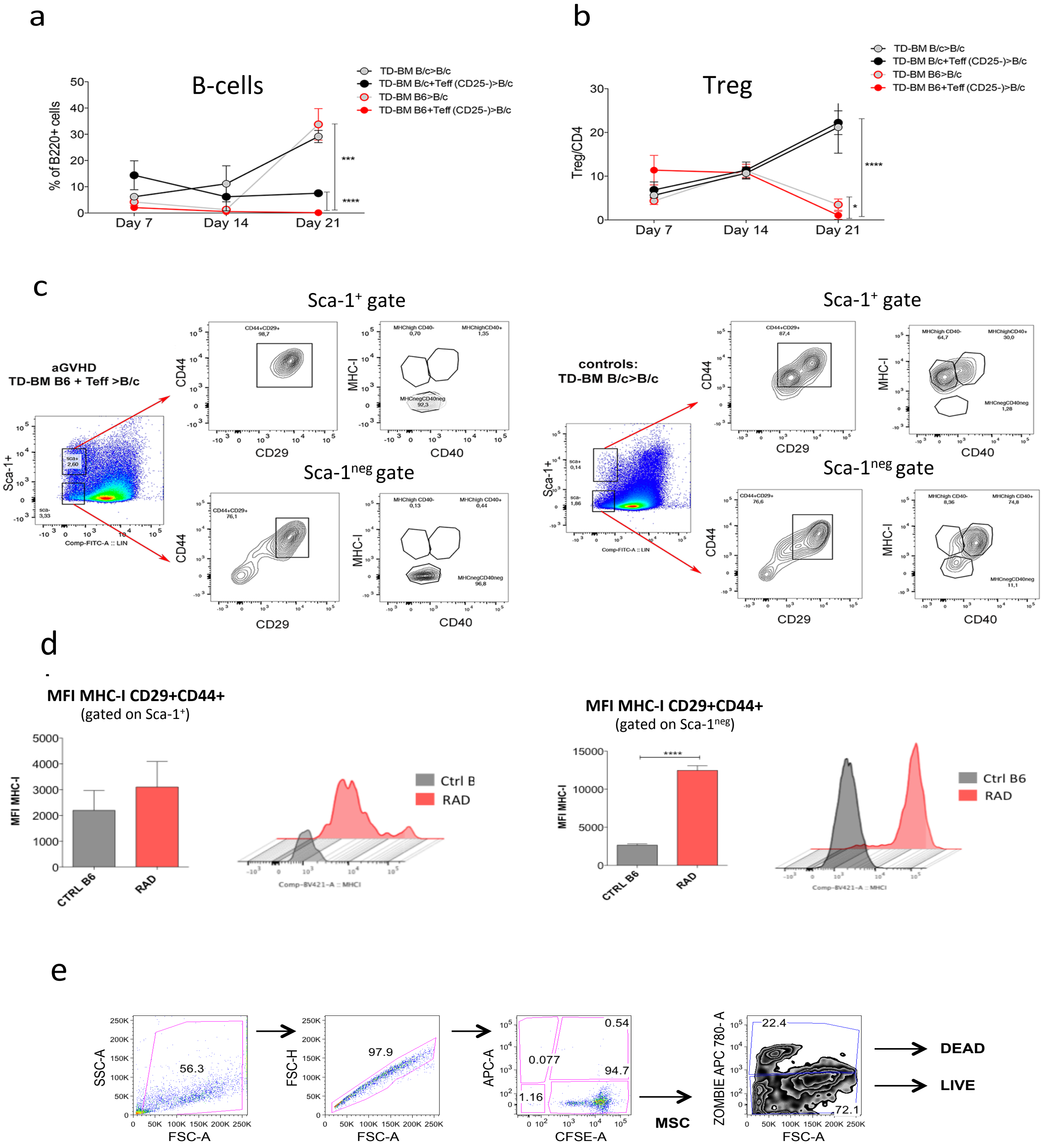

MHC-I expression on Sca+ and Sca- BM-MSCs (CD29+CD44+) upon lethal irradiation.
a. B-cells and **b.** Tregs at days 7, 14, and 21 post-allogeneic BMT compared to controls (n = 5 per group). ***p < 0.001, compared by Student's *t* test. **c.** Representative gating strategy showing MHC^{high}CD40+, MHC^{high}CD40- and of MHC^{neg}CD40+ BM-MSCs in the Sca-1+ gate and Sca-1^{neg} gate of Lin-CD44+CD29+ BM-MSCs in aGVHD vs controls mice. The analysis was performed at 14 days post-transplantation. **d.** Cumulative data displaying the increase MFI of MHC-I in irradiated compared to non irradiated (control) mice. Representative histograms are also shown.

****p < 0.0001, compared by Student's *t* test. **e.** representative gating strategy of the cytotoxicity assay performed on MSC incubated with splenocytes freshly isolated from aGVHD or control mice.

Supplementary Table 1. Patients characteristics.

Patient	day of biopsy post-transplant	underlying disease	age at transplantation/sex	ATG	immunosuppression	aGVHD	cGVHD	IHC Pax5	IHC CD40
#1	d28	AML	59/m	x	Cyclosporine, Cellcept	Grade III		<1%	1 (myelo)
#2	d26	AML	45/m		Cyclosporine, MTX	Grade III		<1%	0
#3	d25	AML	57/f	x	Cyclosporine, Cellcept			6%	1 (myelo)
#4	d21-28	T-NHL+tAML	71/f	x	Cyclosporine, Cellcept			21%	2 (myelo + stroma)
#5	d21	AML	72/m	x	Cyclosporine, Cellcept		Grad III	<1%	0
#6	d24	Ph+ ALL	58/f		Cyclosporine, MTX	Grade I		5%	2 (myelo)
#7	d28	AML	43/f		Cyclosporine, MTX	Grade II		3%	1 (myelo)
#8	d28	AML	62/f	x	Cyclosporine, Cellcept			15%	2 (myelo + stroma)
#9	d28	AML	57/m	x	Cyclosporine, Cellcept	Grade II		8%	1 (myelo + stroma)
#10	d22	AML	58/f	x	Cyclosporine, Cellcept			12%	1 (myelo + stroma)
#11	d28	ALL	23/m		Cyclosporine, MTX	Grade II		<1%	1 (myelo)
#12	d28	AML	60/m	x	Cyclosporine, Cellcept	Grade II		<1%	1 (myelo)

Supplementary Table 2. List of antibodies.

Fluorescence	Antigen	Clone	Manufacturer
**BM ANALYSIS**			
**APC-eFluor 780**	**B220**	RA3-6B2	eBioscience
**APC**	**CD43**	S7	BD
**PECy7**	**IgM**	R6-60.2	BD
**PE**	**CD24**	30-F1	BD
**PE**	**B220**	RA3-6B2	BD
**FITC**	**BPI**	F6354	eBioscience
**SPLENIC B-CELL COMPOSITION**			
**FITC**	**CD43**	eBioR2/60	eBioscience
**APC-Cy7**	**B220**	RA3-6B2	Biolegend
**APC**	**CD93**	AA4.1	eBioscience
**PE**	**CD23**	B3B4	eBioscience
**FITC**	**IgD**	11-26c	eBioscience
**PerCP**	**CD21/35**	7E9	Biolegend
**APC**	**CD3**	145-2C11	BD
**PE**	**CD11c**	N418	Tonbo Biosciences
**Pe-Cy7**	**CD11b**	M1/70	Tonbo Biosciences
**APC -e-fluor780**	**Gr-1**	RB6-8C5	eBioscience
**T-CELL ANALYSIS**			
**FITC**	**CD25**	eBio7D4	eBioscience
**FITC**	**TNFα**	MP6-XT22	BD
**PE**	**OX40**	OX86	Biolegend
**PerCPCy5.5**	**Foxp3**	FJK-16S	eBioscience
**PE-Cy7**	**CD4**	RM4-5	Tonbo Biosciences
**APC**	**IFNγ**	XMG1.2	eBioscience
**APC-eFluor780**	**CD45.2**	L104	Biolegend
**APC-eFluor780**	**CD45.1**	A20	eBioscience

Fluorescence	Antigen	Clone	Manufacturer
**BM-MSC ANALYSIS**			
**FITC**	**CD45**	30-F11	Tonbo Biosciences
**FITC**	**CD31**	390	eBioscience
**FITC**	**TER119**	Ter119	Invitrogen
**FITC**	**CD34**	RAM34	BD
**FITC**	**cKIT**	2B8	eBioscience
**APC**	**SCA-1**	D7	BD
**APC-Cy7**	**CD44**	IM7	BD
**BV421**	**MHC-I**	AF6-88.5	BD
**BV605**	**CD73**	TY/11.8	Biolegend
**BV711**	**MHC-II**	M5/114.15.2	BD
**PE**	**CD40**	3/23	BD
**BV421**	**CD40**	3/23	BD
**PE-Cy7**	**CD29**	eBioHMb1-1	eBioscience

Antigen	Type	dilution	Manufacturer
**Immunohistochemistry (IHC)**			
**PAX-5 (ab109443)**	monoclonal anti-human	1:1000	Abcam
**CD40 (ab13545)**	polyclonal anti-human and mouse	1:100	Abcam
**iNOS (ab15323)**	polyclonal anti-mouse	1:100	Abcam
**PD-L1 (D5V3B)**	monoclonal anti-mouse	1:200	Cell Signaling
**OX40L (RM134L)**	monoclonal anti-mouse	1:100	BD

**Co-immunofluorescence**			
**B220 (9F14)**	monoclonal anti-mouse	1:50	Genetex
**CD40 (ab13545)**	polyclonal anti-human and mouse	1:100	Abcam
**OX40L (RM134L)**	monoclonal anti-mouse	1:100	BD
**CD146 (ab49492)**	monoclonal anti-human	1:25	Abcam
**anti-nestin PE-conjugated**	polyclonal	1:100	Invitrogen
